# Diagnostic challenges in complicated case of glioblastoma

**DOI:** 10.3389/pore.2024.1611875

**Published:** 2024-10-29

**Authors:** Tatiana Aghova, Halka Lhotska, Libuse Lizcova, Karla Svobodova, Lucie Hodanova, Karolina Janeckova, Kim Vucinic, Martin Gregor, Dora Konecna, Filip Kramar, Jiri Soukup, David Netuka, Zuzana Zemanova

**Affiliations:** ^1^ Center of Oncocytogenomics, Institute of Medical Biochemistry and Laboratory Diagnostics, General University Hospital and 1st Faculty of Medicine of Charles University in Prague, Prague, Czechia; ^2^ Laboratory of Genomics and Bioinformatics, Institute of Molecular Genetics of the Czech Academy of Sciences, Prague, Czechia; ^3^ Laboratory of Integrative Biology, Institute of Molecular Genetics of the Czech Academy of Sciences, Prague, Czechia; ^4^ Department of Neurosurgery, 1st Faculty of Medicine of Charles University and Military University Hospital Prague, Prague, Czechia; ^5^ Department of Pathology, 1st Faculty of Medicine of Charles University and Military University Hospital Prague, Prague, Czechia; ^6^ The Fingerland Department of Pathology, Charles University, Faculty of Medicine in Hradec Králové and University Hospital Hradec Králové, Hradec Králové, Czechia; ^7^ Department of Pathology, Charles University, First Faculty of Medicine and General University Hospital in Prague, Prague, Czechia

**Keywords:** diagnostics, cytogenomics, MLPA, I-FISH, aCGH/SNP, gene panel, WGS

## Abstract

Glioblastoma is the commonest primary malignant brain tumor, with a very poor prognosis and short overall survival. It is characterized by its high intra- and intertumoral heterogeneity, in terms of both the level of single-nucleotide variants, copy number alterations, and aneuploidy. Therefore, routine diagnosis can be challenging in some cases. We present a complicated case of glioblastoma, which was characterized with five cytogenomic methods: interphase fluorescence *in situ* hybridization, multiplex ligation-dependent probe amplification, comparative genomic hybridization array and single-nucleotide polymorphism, targeted gene panel, and whole-genome sequencing. These cytogenomic methods revealed classical findings associated with glioblastoma, such as a lack of *IDH* and *TERT* mutations, gain of chromosome 7, and loss of chromosome 10. At least three pathological clones were identified, including one with whole-genome duplication, and one with loss of 1p and suspected loss of 19q. Deletion and mutation of the *TP53* gene were detected with numerous breakends on 17p and 20q. Based on these findings, we recommend a combined approach to the diagnosis of glioblastoma involving the detection of copy number alterations, mutations, and aneuploidy. The choice of the best combination of methods is based on cost, time required, staff expertise, and laboratory equipment. This integrated strategy could contribute directly to tangible improvements in the diagnosis, prognosis, and prediction of the therapeutic responses of patients with brain tumors.

## Introduction

Glioblastoma is the commonest primary malignant brain tumor, and has a very poor prognosis [1] and short overall survival [2]. Historically, glioblastoma has been routinely diagnosed and classified based on its histopathology, according to a microscopic evaluation and its clinical behavior [3–5]. In recent decades, new diagnostic tools have been introduced that produce diagnostic and prognostic information that is more accurate, including interphase fluorescence *in situ* hybridization (I-FISH), multiplex ligation-dependent probe amplification (MLPA), and comparative genomic hybridization array and single-nucleotide polymorphism (aCGH/SNP) analyses [6]. Since 2008, with the introduction of next-generation sequencing (NGS) and its application to numerous diagnostic methods (e.g., whole-genome sequencing [WGS] and targeted gene panels), the simultaneous sequencing of several markers associated with tumors has become possible. The World Health Organization (WHO) and the European Association of Neuro-Oncology (EANO) recently implemented molecular diagnostic guidelines for the classification, treatment, and follow-up of glioblastoma [7, 8].

According to the current classification, glioblastoma (WHO grade 4) is defined by the following molecular features: lack of mutations in isocitrate dehydrogenase (*IDH*) genes, absence of G34R/V or K27M mutations in *H3F3A/H3F3B*, presence of mutations in the telomerase reverse transcriptase *(TERT)* gene promoter, amplification of the epidermal growth factor receptor (*EGFR)* gene, and the combined gain of chromosome 7 and loss of chromosome 10 [7, 8].

Another characteristic feature of glioblastoma is its high intra- and intertumoral heterogeneity [9, 10]. The level of intratumor genetic diversity has been shown to correlate with cancer progression and mortality [11–14]. Many types of genetic heterogeneity are associated with cancer, including single-nucleotide variants (SNVs), copy-neutral loss of heterozygosity (CN-LOH), copy number alterations (CNAs), and aneuploidy [15]. Therefore, the diagnosis of glioblastoma often poses significant challenges, requiring the integration of multiple advanced cytogenomic techniques to achieve diagnostic clarity.

Here, we present a complex case of a patient with glioblastoma. Samples obtained during routine neurosurgery were analyzed with WGS and several other cytogenomic approaches used in the routine diagnosis of brain tumors. A comparison of the results allowed us to define the advantages and limitations of these methods and to discuss any ambivalent results. This overview provides not only theoretical, but also practical insight into the complexity of glioblastoma diagnosis.

## Materials and methods

### Biopsy processing

The patient with glioblastoma underwent neurosurgical resection at the Department of Neurosurgery, Military University Hospital, Prague, Czech Republic. The patient gave his written consent for his biological material to be used for research purposes, in accordance with the ethical standards of the local ethic committees (Ref. No. 108/15-33/2020). Two biopsies were taken from a similar region of the brain tumor during the routine neurosurgical procedure, for diagnostic and experimental purposes. The first was used for the histopathological diagnosis of the tumor mass. The second biopsy sample was immersed in phosphate-buffered saline (PBS) containing 1% heparin (Zentiva, Prague, Czech Republic) and transferred to the Center of Oncocytogenomics, General University Hospital, Prague, Czech Republic) for further analysis. The biopsy sample was homogenized at medium speed for 45 s with a homogenizer (Minilys, Bertin Instruments, Montigny-le-Bretonneux, France), and then divided into two parts. The first part was fixed and used for I-FISH (see I-FISH protocol). The other part was centrifuged (24,000 × *g*, 5 min, 4°C) and the pellet was used for isolating genomic DNA (gDNA). The patient’s peripheral blood, taken after the procedure, was placed in Vacuette 2 mL K3-EDTA tubes (Dialab, Prague, Czech Republic). The gDNA was isolated and used as a control to eliminate germline variants and copy number changes.

### gDNA isolation and quantification

The gDNA was isolated from the tumor sample with the DNeasy Blood and Tissue Kit (Qiagen, Valencia, CA, United States), according to the manufacturer’s protocol. The GenElute Blood Genomic DNA Kit (MERC, Darmstad, Germany) was used to isolate the peripheral blood gDNA, according to manufacturer’s instructions.

The quality and quantity of gDNA were assessed with a spectrophotometer (NanoDrop 2000, Thermo Fisher Scientific, Waltham, MA, United States) and a fluorometer (Qubit 4.0, Life Technologies, Carlsbad, CA, United States). The quality of the gDNA was confirmed on 1% agarose gel, after visualization with a gel imaging system (Essential V6, Uvitec Cambridge, Cambridge, United Kingdom).

### Interphase fluorescence *in situ* hybridization (I-FISH)

The homogenized tissues in PBS containing heparin were processed further with standard cytogenetic procedures (hypotonia, fixation in methanol/acetic acid). Microscopy samples for I-FISH were prepared from fixed cell suspensions. Dual-color interphase FISH was performed with the DNA probes Vysis LSI 1p36 SpO/1q25 SpG, LSI 19q13 SpO/19p13 SpG, LSI PTEN/CEP 10, LSI TP53/CEP 17, LSI 13 (RB1)/13q34, CEP X SpO/Y SpG (Abbott Molecular, Des Plaines, IL, United States), XL EGFR amp, XL 6q21/6q23/6cen, XL CDKN2A (MetaSystems, Altlussheim, Germany), and MGMT-20-OR (Empire Genomics, Williamsville, NY, United States), according to the manufacturers’ recommendations, to detect the biomarkers for glioblastoma categorization and to confirm the findings of the other methods. The slides were analyzed with an fluorescence microscope (Axio Imager Z2, Zeiss, Oberkochen, Germany) by two independent observers. The cut-off values were defined as 5% for deletions and 2.5% for gains, as described in a previous study [16].

### Multiplex ligation-dependent probe amplification (MLPA)

The MLPA probe mixes (MRC Holland, Amsterdam, Netherlands) p370 BRAF-IDH1-IDH2 (version C1), p150 Glioma2 (version D3), and p088 Oligodendroglioma 1p-19q (version D1) were used to detect CNAs and selected variants of the *IDH1*, *IDH2*, and *BRAF* genes. The methylation-specific MLPA probe mix ME012 MGMT-IDH1–IDH2 (version A1) (MRC Holland, Amsterdam, Netherlands) was used to detect the methylation of the *MGMT* promoter and selected mutations in the *IDH* genes, according to the manufacturer’s instructions. The PCR amplicons were analyzed with the SeqStudio Genetic Analyzer System (Applied Biosystems, Waltham, MA, United States). The acquired fragmentation data were analyzed with Coffalyser.Net (MRC Holland). MLPA probes with scores of ≥1.3 were deemed to show gains/amplifications, and those with scores of 1.15–1.29 were deemed to be the suspected gains/amplifications. MLPA probes with scores of ≤0.7 were deemed to show losses/deletions and those with scores of 0.71–0.85 were deemed to show suspected losses.

### Comparative genomic hybridization array and single-nucleotide polymorphism (aCGH/SNP)

A microarray analysis (aCGH/SNP) was performed with the SurePrint G3 Human CGH Microarray Kit, 4 × 180K (Agilent Technologies, Santa Clara, CA, United States) to detect unbalanced chromosomal changes and CN-LOH. The final product was scanned with a microarray scanner system (G2565CA, Agilent Technologies) and analyzed with the CytoGenomics 5.2.0.20 software (Agilent Technologies). Unbalanced chromosomal abnormalities of >350 kb and CN-LOH regions of >10 Mb are reported.

### Targeted gene panel

The Archer VariantPlex Solid Tumor Kit (IDT, Coralville, IA, United States), which detects SNVs and CNAs in 67 genes associated with solid tumors, was used to analyze the tumor samples, according to the manufacturer’s recommendations. Final quantification was performed with the KAPA Library Quantification Kit (Roche, Basel, Switzerland), followed by sequencing with the NextSeq 500/550 Mid Output Kit v2 (300 Cycles) (Illumina, San Diego, CA, United States). The quality of runs was examined with Sequencing Analysis Viewer v2.4.7 (Illumina) and FASTQC v0.11.9 [17]. The analysis was performed in Archer software v7 (IDT). Only variants that met our criteria (allele frequency > 5%, quality score > 1,000, depth > 500) were considered further. The variants from the peripheral blood were compared with the variants from the glioma samples and only somatic variants were analyzed further (Supplementary Table S4).

### Whole-genome sequencing (WGS)

DNA was fragmented with an ultrasonicator (M220, Covaris, Woburn, MA, United States) with the program set up for 500–600-bp fragments. The fragment length distribution was controlled with an Agilent Bioanalyzer 2100 (High Sensitivity DNA Kit) (Agilent). Sequencing libraries were prepared from the fragmented DNA (40 ng) with the NEBNext Ultra II DNA Library Prep Kit for Illumina (New England BioLabs, Ipswich, MA, United States). NEBNext Multiplex Oligos for Illumina (Dual Index Set 1) (New England BioLabs, Ipswich, MA, United States) was used to barcode the libraries. The final PCR amplification of the library included six cycles and the products were cleaned up with SPRIselect beads (Beckman Coulter, Brea, CA, United States). The library length profiles and concentrations were controlled with the Agilent Bioanalyzer (High Sensitivity DNA Kit) and fluorometer (Qubit 2.0, Life Technologies, Carlsbad, CA, United States), respectively. The libraries were mixed in equimolar ratios and commercially sequenced on a sequencing system (NovaSeq 6000, Illumina) to obtain a minimum of 5000 paired-end reads (150 bp × 150 bp).

The data analysis workflow consisted of quality control, alignment, and somatic variant calling. The analysis was performed with the nf-core/sarek pipeline v2.7.1 [18] with the default options, unless otherwise stated. Read quality was assessed with FastQC v0.11.9 [17], and the reports were combined with MultiQC v1.12 [19]. The reads were aligned to the GRCh38 human reference genome [20]. The somatic variant calling of SNVs was performed with GATK MuTect2 v4.1.7.0, Strelka v2.9.10, and the best-practice modification of Strelka, called “StrelkaBP” [21]. Structural variants (SVs) were determined with Manta v1.6.0 [22]. CNVs, LOH, and neutral events were detected with both Control-FREEC v11.6 [23] and ASCAT v2.5.2 [24]. ASCAT was used to estimate ploidy and tumor heterogeneity. SNVs were annotated with snpEff v4.3t [25] and Ensembl Variant Effect Predictor (VEP) v99.2 [26], whereas SVs were only annotated with VEP. Both tools used the ENSEMBL annotation file GRCh38 v99. Data manipulation, report generation, and the creation of several plots were performed in R v4.1.3 [27]. Circos v0.69.8 software [28] was used to visualize the SV and CNV data in circos plot.

## Results

### Case presentation

In December 2020, a 65-year-old male patient experienced an epileptic seizure with postictal right-sided hemiparesis and was diagnosed with glioblastoma of the left parietal lobe. Surgery was postponed because the patient became infected with SARS-CoV-2. Two months later, the patient underwent subtotal resection of the tumor, and minimal residue was detected on a postoperative MRI scan. The patient’s recovery was uncomplicated, but slight right-sided hemiparesis persisted. After the results of the histological analysis were received—glioblastoma, *IDH*-wildtype – concomitant chemoradiotherapy was recommended and begun at the end of February 2021. However, the tumor had recurred at the first check-up, 4 months after surgery. The Karnofsky performance score of the patient was 50–60. Given the rapid decline in the patient’s condition, further surgery was not recommended and adjuvant chemotherapy with temozolomide was suggested as the best option. Despite oncological treatment, the tumor continued to grow. The patient died in March 2022.

A microscopic examination showed brain tissue infiltrated by a highly cellular diffuse glioma, with prominent nuclear atypia and high proliferative activity (5–6 mitoses per high-power field, including atypical mitoses). Foci of microvascular proliferation and areas of necrosis were present, with a palisade-like arrangement of tumor cells at the periphery. Immunochemistry revealed that the nuclear expression of *ATRX* was retained in all the tumor cells, there was no R132H mutation in the *IDH1* gene, loss of p53 immunoreactivity and the Ki67 index was up to 25% in highly proliferative areas (data not shown).

### Cytogenomic findings

**FIGURE 1:**
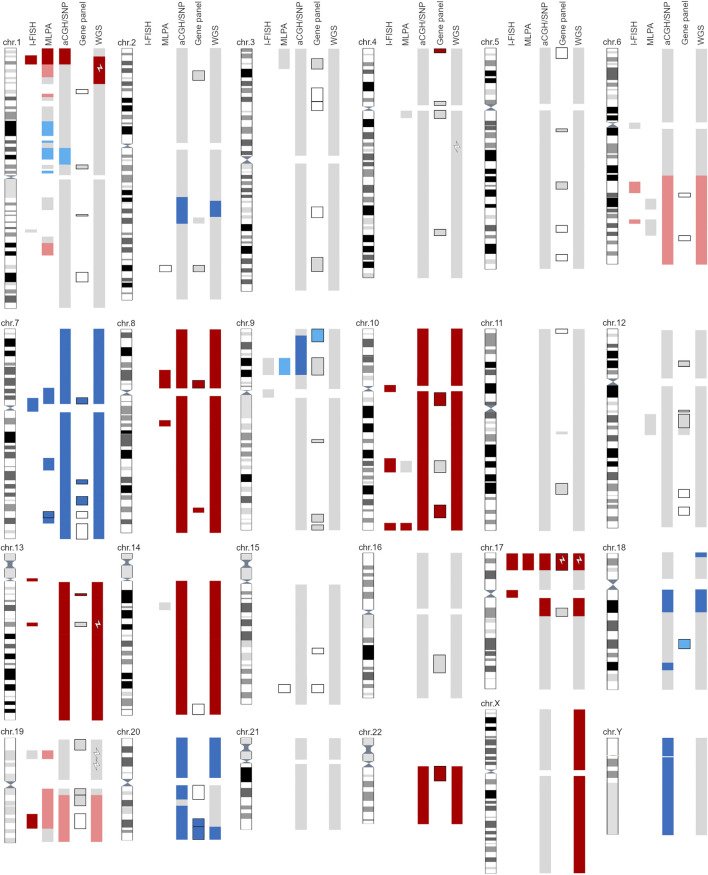
Graphic representation of all reported findings with five cytogenomics methods. Light gray—area with normal findings investigated with selected method. Dark blue—gain/amplification of designated area; light blue—suspected gain of marked area (at the border of detection limit). Dark red—loss/deletion of designated area; light red—suspected loss of marker area (at the border of detection limit). Black outlined areas indicate detection of SNVs with gene panel and/or MLPA. White lightning bolts represent positive gene mutations detected with the selected method. For detailed information, see Supplementary Material.

The genetic aberrations specific for glioblastoma were detected with a combination of cytogenomic methods, and the diagnosis was confirmed with histopathological analyses (Figure 1, Supplementary Tables S1–S7). No mutation of *IDH1/IDH2* was identified; nor was the G34R/V or K27M mutation in *H3F3A* or any mutation in the *TERT* promoter detected. Gain of chromosome 7 (including the *EGFR* gene) was observed (trisomy–hexasomy on I-FISH, and up to eight copies were detected with WGS). Monosomy of chromosome 10 was observed with I-FISH, aCGH/SNP, and WGS, although MLPA and the gene panel indicated an intact *PTEN* gene (Figure 1, Supplementary Table S2). Methylation of the *MGMT* promoter was not confirmed (Supplementary Table S2).

As well as glioblastoma-specific cytogenomic aberrations, additional SNAs, CNVs, SVs, and aneuploidy were detected. I-FISH and WGS detected at least three pathological clones, one of which was near-tetraploid (5%–15% interphase nuclei, depending on the selected probe; ploidy = 3.83). I-FISH detected the deletion of 1p36 and 19q13 (Figure 1, Supplementary Table S1). Other methods (MLPA, aCGH/SNP, and WGS) only identified the deletion of 1p36, and a suspected deletion of 19p (below the cut-off limit; Figure 1, Supplementary Tables S2, S3, S7).

In the near-tetraploid clone, two types of chromosomal losses were identified based on the WGS results: 1) chromosome losses with a homozygous allelotype (two copies instead of four), such as chromosomes 8, 10, 13, 14 and 22; and 2) chromosome losses with a heterozygous allelotype (three copies instead of four), such as the long arm of chromosome 6 and the loss of chromosomes 19 and 21. The same mechanism was also observed for gains (five chromosomes instead of four), such as the gain of one copy each of chromosomes 1, 9, 13 and 18 (Supplementary Figure S1).

The loss of 17p was confirmed with all the methods used. Moreover, the I-FISH analysis revealed monosomy 17 in a small cell clone (Figure 1, Supplementary Table S1). The pathogenic variant R196* of the *TP53* gene was subsequently detected with the gene panel and WGS (Supplementary Tables S4, S6).

The WGS data revealed seven SNVs that were classified as pathogenic or likely pathogenic (Tiers I/II) and 13 variants of unknown significance (Tier III) (Supplementary Table S6). WGS also confirmed SVs on nearly all chromosomes, except chromosomes 13 and 14. Among these, deletions (n = 88) were most frequent, followed by breakends (BNDs; n = 80), duplication events (n = 42), and insertions (n = 4). Two chromosomes showed the most BNDs: the short arm of chromosome 17 (17p13.1 and 17p12) with 14 BNDs (10 BNDs and 2 interchromosomal translocations) and chromosome 20 with 29 BND events (19 BNDs and 5 interchromosomal translocations) (Supplementary Figures S2, S3). Detailed results of the SV analysis are given in the Supplementary Figures S1–S3 and Supplementary Tables S6, S7.

## Discussion

The findings in glioblastoma patients are usually very complex, ranging from clinically significant aberrations to complex structural rearrangements that are detectable only with methods such as WGS. In this study, we represent one case that was selected as an ideal candidate for methods presentation due to the complexity of his findings, which thoroughly tested our laboratory methodologies.

One of the most important factors influencing the prognosis of glioblastoma patients is CNAs [29], followed by SNVs [30]. Recent data have shown that gene fusions and rearrangements are also important drivers in some brain tumors [6, 31]. All methods compared in this study detected CNAs, although with considerable differences in the numbers detected, based on their detection limits (Figure 1, Table 1, Supplementary Material). I-FISH and MLPA required several probes or kits to detect all the CNAs necessary for a molecular diagnosis of glioblastoma (Table 1). Other methods (aCGH/SNP, gene panel, WGS) detected all the important CNAs in one assay. However, in general, NGS methods (gene panels, WGS) are not optimal for large CNAs, such as the gain or deletion of whole chromosomes or chromosomal arms [32]. In our patient, the gene panel detected partial deletions of chromosomes 10 and 13, but not monosomies of the whole chromosomes (Figure 1). CNA-recognition software is based on the coverage of amplicons and the detection of outliers after the appropriate normalization step. Therefore, it is crucial to analyze several samples together, ideally with reference samples as the negative controls, and thereby improve the detection of large CNAs with gene panels.

**TABLE 1:** Comparison of different cytogenomic methods for glioblastoma diagnosis.

	I-FISH	MLPA	aCGH/SNP	Gene panel	WGS
Input material	200 nuclei	15–450 ng DNA	1,000 ng DNA	1–100 ng DNA	5–100 ng DNA
PCR	No	Yes	No	Yes	Yes/no
Sequencing	No	Capillary electrophoresis	No	Illumina/others	Illumina/others
Analysis					
Wet-laboratory time	<2 days	<2 days	2–3 days	3–5 days	3–5 days
Analysis time	20 min	10 min	30 min	3 h	>3 h^a^
Analysis software	Fluorescence microscope, ISIS (MetaSystems)	Coffalyser (MRC Holland)	CytoGenomics (Agilent); GenomeStudio Software (Illumina); BlueGnome (Illumina); CytoSure Interpret Software (OGT)	Archer Software (ITD); BaseSpace (Illumina); SOPHiA DDM Platform (Sophia Genetic)	Bioinformatic pipelines
Time for interpretation of results	5 min	30 min	2 h	10 min–2 h	>2 h^a^
SNVs/mutations					
*IDH1*	No	Yes	No	Yes	Yes
*IDH2*	No	Yes	No	Yes	Yes
*BRAF*	No	Yes	No	Yes	Yes
*EGFR*	No	No	No	Yes	Yes
*TP53*	No	No	No	Yes	Yes
*H3F3A*	No	No	No	Yes	Yes
*TERT* promoter	No	Yes (ME012)	No	Yes	Yes
CNAs					
1p/19q co-deletion	Yes	Yes	Yes	Yes	Yes
chr 7 gain (*EGFR*)	Yes	Yes	Yes	Yes	Yes
chr 9 loss (*CDKN2A/CDKN2B*)	Yes	Yes	Yes	Yes	Yes
chr 10 loss (*PTEN, MGMT*)	Yes	Yes	Yes	Yes	Yes
*ATRX* loss	Yes	Yes (P013)	Yes	Yes	Yes
Polyploidy	Yes	No	No	No	Yes
CN-LOH	No	No	Yes	No	Yes
SVs	No	No	No	Yes (only specific SV)	Yes

The criteria for comparison were the input material and laboratory equipment, time for wet laboratory testing, time for analysis and interpretation, detections of the single-nucleotide variants (SNVs), copy number alterations (CNAs), copy-neutral loss of heterozygosity (CN-LOH), and structural variants (SVs).

^a^ It can take from several hours up to a few days, depending on the data, analyst expertise and bioinformatic pipeline.

I-FISH is the standard diagnostic method for detecting the unbalanced whole-arm translocation t(1; 19)(q10; p10), which is a typical finding in oligodendroglial tumors [7]. This rearrangement results in the whole arm 1p/19q co-deletion, which is used as an important diagnostic marker [33]. However, FISH probes only allow targeted detection of the loss of specific chromosomal regions 1p36 and 19q13 [34, 35] but do not provide information on the extent and origin of these deletions. Thus, they can lead to false-positive findings. Similarly in our patient, I-FISH detected deletion of 1p36 and 19q13, i.e., a suspected 1p/19q co-deletion. However, MLPA, aCGH/SNP and WGS analyses revealed only partial loss of the short arm of chromosome 1 (1p35p36) and the hole long arm of chromosome 19 (Figure 1), so the origin of the deletions was likely different from the recurrent t(1; 19)(q10; p10) translocation. These losses are a relatively common finding in astrocytic tumors [36, 37]. According to published data, the frequency of false-positive FISH findings of 1p/19q co-deletion in diffuse astrocytomas is estimated to be approximately 3.6% [38]. Therefore, in *IDH*-mutated gliomas, it is beneficial to investigate the 1p/19q co-deletion with two independent methods to refine the type of these deletions, as recommended by Brandner [39]. The loss of chromosome 10 was observed, although MLPA and the gene panel indicated an intact *PTEN* gene. This ambivalent result may be caused by the presence of small polyploid clones. The presence of more than two copies of chromosome 10 may affect the relative ratio calculated with these methods. The LOH on the short arm of chromosome 17 frequently occurs in human cancers, often including the region containing the *TP53* tumor suppressor gene (17p13.1; [1]). *TP53* mutations or deletions are observed in 23%–28% of patients with primary glioblastoma [40, 41]. The complete loss of the *TP5*3 gene is commonly associated with t(17; 20) reciprocal translocations in glioblastoma [42]. Our data confirms the translocation breakpoints at loci on 17p and 20q (Supplementary Figure S3). *TP53* alterations have been shown to be mutually exclusive with the amplifications of the *MDM* family genes *(MDM1/2/4*) and *CDKN2A* [40], which is consistent with our findings. The pathogenic variant *TP53* R196*, which was detected in our patient, was previously described by Shiraishi et al. [43].

Concurrent *TP53* mutations are twice as common in the presence of whole-genome duplication (WGD), although *TP53* dysfunction is not an obligatory event for WGD [44]. One of the most important findings that caused ambiguous results was the presence of several clones, one of which was a small near-tetraploid clone (7% of cells). Only two methods (I-FISH, WGS) could reliably detect aneuploidy. WGD leads to tetraploidy and is a common macroevolutionary event occurring early in tumorigenesis [44, 45]. Its frequency is reported to range from 11% [45, 46] to 25% in glioblastoma [47]. WGD has been associated with several molecular and clinical features, including a higher mutational burden, increased proliferation signatures, worse overall survival, and resistance to radiation therapy and chemotherapy [44, 46, 48]. WGD may also contribute to intratumor heterogeneity. Intratumoral heterogeneity has been well documented with single-cell RNA-sequencing experiments, which revealed cross-talk among four different cellular states [49, 50]. We were unable to assign our finding to these states, but at least three clones were observed based on the I-FISH and WGS results (Supplementary Figure S1). Moreover, the same pathological clone, discovered with I-FISH in diploid cells, was also present after its duplication in near-tetraploid (Supplementary Figure S1). The remaining methods (MLPA, aCGH/SNP, and gene panel) detected only the changes present in most tumor cells, and in targeted chromosomal regions (such as the long arm of chromosome 6 and the short arm of chromosome 9), so the presence of several clones with various findings led to inconclusive results.

The diagnostic routine for solid tumors currently requires that the results be delivered within 14 days of the receipt of the sample [41]. The time required for the laboratory processing range from several hours or days (I-FISH, MLPA) to ≥3 days (aCGH/SNP, gene panels, WGS), depending on the number of samples, the amount of sample delivered, and the complexity of each sample (Table 1). In terms of the time required to interpret the results, the methods that are based on gDNA analyses and that can only detect the most abundant aberrations (such as MLPA and gene panels) are less time-consuming. The time required to interpret an I-FISH analysis can be influenced by the number of pathological clones present in the sample. The challenge of WGS is the expertise and time required to interpret the full spectrum of SNAs, CNVs, and SVs [51]. Analysis and interpretation can take several hours or up to several days, depending upon the quality of the data and the number of SNVs and CNVs. Another important component of diagnostic testing is the cost of the analysis performed, which may be linked to insurance reimbursement [52]. The most economically efficient procedure is MLPA, followed by I-FISH and aCGH/SNP. Gene panels are more cost-effective than WGS, because WGS inherently requires substantially more sequencing capacity than gene panels, and thus is more expensive. Protocols for WGS at very low depth have recently been established to reduce costs (e.g., [53]). The availability or selection of a sequencing instrument with high throughput capacity can dramatically reduce the cost of methods based on NGS. However, for small laboratories, the number of samples could also be a limitation. The time required to collect sufficient samples for NGS-based approaches or even aCGH/SNP can prolong the delivery of the results to neurosurgeons and increase the sequencing cost.

The methods compared in our study detected CNAs, SNVs, SVs, and aneuploidy, either alone or in combination. Nevertheless, these methods cannot detect the exact number of pathogenic clones, microsatellite instability, the expression of genes, and/or translocations. There are many technologies available today, including Single-cell DNA sequencing, Single-cell RNA sequencing, epigenetic profiling, optical genome mapping and circulating and tumor cell enumeration, that can together provide a comprehensive “snapshot” of an individual cancer. However, this is not economically feasible for routine practice yet.

The prevailing diagnostic method for glioblastoma is based on an investigation of several selected aberrations in a relatively short period so that the patient’s treatment can begin as soon as possible. The treatment approach recommended by the National Comprehensive Cancer network (NCCN) guidelines for glioblastoma patients with good performance status (PS) includes maximal surgical removal, followed by radiotherapy and chemotherapy with temozolomide and/or participation in clinical trial [54]. The genomic findings in this case, particularly the detection of *TP53* mutations, *EGFR* amplification, and chromosomal alterations such as gain of chromosome 7 and loss of chromosome 10, carry important implications for treatment. The p53 pathway (TP53/MDM2/P14^arfç^) and PI3K/AKT/mTOR pathway (activated by *EGFR* gene amplification) are the main signalling pathways involved in glioblastoma tumorigenesis and are associated with resistance to standard treatments [55, 56]. Therapies targeting the *TP53* gene aim to restore normal level of p53 tumor suppressor protein through inhibition of p53/MDM2 complex [57] or MDM2 itself [58]. Similarly, *EGFR* amplification opens the potential for targeted therapies, including three generations of EGFR inhibitors [59, 60] or EGFR antibodies [61]. However, the heterogeneity observed in the tumor complicates treatment decisions, as the presence of multiple clones with varying genetic profiles suggests that single-target therapies may not be sufficient. Comprehensive molecular profiling in glioblastoma cases, as demonstrated in this study, is crucial for tailoring personalized treatment strategies and potentially improving patient outcomes.

We have shown that although the presence of several pathological clones and aneuploidy can complicate the examination of the sample, it is feasible to use a combination of modern molecular diagnostic methods. Based on the findings presented here, a combined approach involving the detection of CNAs (I-FISH, MLPA, aCGH/SNP), mutations (MLPA, gene panel), and aneuploidy (I-FISH) is recommended for routine diagnosis. The great promise for the future looks to be WGS, which can detect the most sensitive SNAs, CNVs and aneuplodies in a single analysis. The choice of the best combination of methods will be based on price, time required, staff expertise, and laboratory equipment available. This integrated strategy could contribute directly to a tangible improvement in the diagnosis, prognosis, and prediction of the therapeutic responses of patients with brain tumors.

## Data Availability

All data generated or analyzed during this study are included in this published article (and in its Supplementary Material).

## References

[1] Rasras S Zibara K Vosughi T Zayeri Z. Genetics and epigenetics of glioblastoma: therapeutic challenges. Clin Cancer Invest J (2018) 7:43–9. 10.4103/ccij.ccij_82_17

[2] Stupp R Mason WP Van Den Bent MJ Weller M Fisher B Taphoorn MJ Radiotherapy plus concomitant and adjuvant temozolomide for glioblastoma. N Engl J Med (2005) 352:987–96. 10.1056/nejmoa043330 15758009

[3] Park SH Won J Kim SI Lee Y Park CK Kim SK Molecular testing of brain tumor. J Pathol Transl Med (2017) 51:205–23. 10.4132/jptm.2017.03.08 28535583 PMC5445205

[4] Perry A Wesseling P. Histologic classification of gliomas. Handbook Clin Neurol (2016) 134:71–95. 10.1016/B978-0-12-802997-8.00005-0 26948349

[5] Pessôa IA Amorim CK Ferreira WAS Sagica F Brito JR Othman M Detection and correlation of single and concomitant *TP53, PTEN*, and *CDKN2A* alterations in gliomas. Int J Mol Sci (2019) 20:2658–20. 10.3390/ijms20112658 31151164 PMC6600458

[6] Horbinski C Ligon KL Brastianos P Huse JT Venere M Chang S The medical necessity of advanced molecular testing in the diagnosis and treatment of brain tumor patients. Neuro-Oncol (2019) 21:1498–508. 10.1093/neuonc/noz119 31276167 PMC6917404

[7] Louis DN Perry A Wesseling P Brat DJ Cree IA Figarella-Branger D The 2021 WHO classification of tumors of the central nervous system: a summary. Neuro-Oncol (2021) 23:1231–51. 10.1093/neuonc/noab106 34185076 PMC8328013

[8] Weller M van den Bent M Preusser M Le Rhun E Tonn JC Minniti G EANO guidelines on the diagnosis and treatment of diffuse gliomas of adulthood. Nat Rev Clin Oncol (2021) 18:170–86. 10.1038/s41571-020-00447-z 33293629 PMC7904519

[9] DeCordova S Shastri A Tsolaki AG Yasmin H Klein L Singh SK Molecular heterogeneity and immunosuppressive microenvironment in glioblastoma. Front Immunol (2020) 11:1402–18. 10.3389/fimmu.2020.01402 32765498 PMC7379131

[10] Patel AP Tirosh I Trombetta JJ Shalek AK Gillespie SM Wakimoto H Single-cell RNA-seq highlights intratumoral heterogeneity in primary glioblastoma. Science (2014) 344:1396–401. 10.1126/science.1254257 24925914 PMC4123637

[11] Almendro V Marusyk A Polyak K. Cellular heterogeneity and molecular evolution in cancer. Ann Rev Pathol (2013) 8:277–302. 10.1146/annurev-pathol-020712-163923 23092187

[12] Maley CC Galipeau PC Finley JC Wongsurawat VJ Li X Sanchez CA Genetic clonal diversity predicts progression to esophageal adenocarcinoma. Nat Genet (2006) 38:468–73. 10.1038/ng1768 16565718

[13] Marusyk A Almendro V Polyak K. Intra-tumour heterogeneity: a looking glass for cancer? Nat Rev Cancer (2012) 12:323–34. 10.1038/nrc3261 22513401

[14] Park SY Gönen M Kim HJ Michor F Polyak K. Cellular and genetic diversity in the progression of *in situ* human breast carcinomas to an invasive phenotype. J Clin Invest (2010) 120:636–44. 10.1172/JCI40724 20101094 PMC2810089

[15] Albertson DG Pinkel D. Genomic microarrays in human genetic disease and cancer. Hum Mol Genet (2003) 12:145–52. 10.1093/hmg/ddg261 12915456

[16] Zemanova Z Michalova K Babicka L Pavlistova L Jarosova M Holzerova M Clinical relevance of complex chromosomal aberrations in bone marrow cells of 107 children with *ETV6/RUNX1* positive acute lymphoblastic leukemia (ALL). Blood (2006) 108:2278. 10.1182/blood.v108.11.2278.2278

[17] Andrews S. FastQC: a quality control tool for high throughput sequence data (2010). Available from: http://www.bioinformatics.babraham.ac.uk/projects/fastqc (Accessed February 2, 2024).

[18] Garcia M Juhos S Larsson M Olason PI Martin M Eisfeldt J Sarek: a portable workflow for whole-genome sequencing analysis of germline and somatic variants. F1000Res (2020) 9:63. 10.12688/f1000research.16665.1 32269765 PMC7111497

[19] Ewels P Magnusson M Lundin S Käller M. MultiQC: summarize analysis results for multiple tools and samples in a single report. Bioinformatics (2016) 32:3047–8. 10.1093/bioinformatics/btw354 27312411 PMC5039924

[20] Schneider VA Graves-Lindsay T Howe K Bouk N Chen HC Kitts PA Evaluation of GRCh38 and *de novo* haploid genome assemblies demonstrates the enduring quality of the reference assembly. Genome Res (2017) 27:849–64. 10.1101/gr.213611.116 28396521 PMC5411779

[21] Kim S Scheffler K Halpern AL Bekritsky MA Noh E Kallberg M Strelka2: fast and accurate calling of germline and somatic variants. Nat Methods (2018) 15:591–4. 10.1038/s41592-018-0051-x 30013048

[22] Chen X Schulz-Trieglaff O Shaw R Barnes B Schlesinger F Kallberg M Manta: rapid detection of structural variants and indels for germline and cancer sequencing applications. Bioinformatics (2016) 32:1220–2. 10.1093/bioinformatics/btv710 26647377

[23] Boeva V Popova T Bleakley K Chiche P Cappo J Schleiermacher G Control-FREEC: a tool for assessing copy number and allelic content using next-generation sequencing data. Bioinformatics (2012) 28:423–5. 10.1093/bioinformatics/btr670 22155870 PMC3268243

[24] Van Loo P Nordgard SH Lingjaerde OC Russnes HG Rye IH Sun W Allele-specific copy number analysis of tumors. Proc Natl Acad Sci USA (2010) 107:16910–5. 10.1073/pnas.1009843107 20837533 PMC2947907

[25] Cingolani P Platts A Wang LL Coon M Nguyen T Wang L A program for annotating and predicting the effects of single nucleotide polymorphisms, SnpEff: SNPs in the Genome of *Drosophila melanogaster* Strain w1118; iso-2; iso-3. Fly (Austin) (2012) 6:80–92. 10.4161/fly.19695 22728672 PMC3679285

[26] McLaren W Gil L Hunt SE Riat HS Ritchie GR Thormann A The Ensembl variant Effect predictor. Genome Biol (2016) 17:122. 10.1186/s13059-016-0974-4 27268795 PMC4893825

[27] The R Development Core Team. R: a language and environment for statistical computing. 2022.

[28] Krzywinski M Schein J Birol I Connors J Gascoyne R Horsman D Circos: an information aesthetic for comparative genomics. Genome Res (2009) 19:1639–45. 10.1101/gr.092759.109 19541911 PMC2752132

[29] Xiao J Jin X Zhang C Zou H Chang Z Han N Systematic analysis of enhancer regulatory circuit perturbation driven by copy number variations in malignant glioma. Theranostics (2021) 11:3060–73. 10.7150/THNO.54150 33537074 PMC7847679

[30] Eckel-Passow JE Decker PA Kosel ML Kollmeyer TM Molinaro AM Rice T Using germline variants to estimate glioma and subtype risks. Neuro-Oncol (2019) 21:451–61. 10.1093/neuonc/noz009 30624711 PMC6422428

[31] Clarke M Mackay A Ismer B Pickles JC Tatevossian RG Newman S Infant high-grade gliomas comprise multiple subgroups characterized by novel targetable gene fusions and favorable outcomes. Cancer Discov (2020) 10:942–63. 10.1158/2159-8290.CD-19-1030 32238360 PMC8313225

[32] Zheng C Miao X Li Y Huang Y Ruan J Ma X Determination of genomic copy number alteration emphasizing a restriction site-based strategy of genome re-sequencing. Bioinformatics (2013) 29:2813–21. 10.1093/bioinformatics/btt481 23962614

[33] Jenkins RB Blair H Ballman KV Giannini C Arusell RM Law M A t(1; 19)(q10; p10) mediates the combined deletions of 1p and 19q and predicts a better prognosis of patients with oligodendroglioma. Cancer Res (2006) 66:9852–61. 10.1158/0008-5472.CAN-06-1796 17047046

[34] Felsberg J Erkwoh A Sabel MC Kirsch L Fimmers R Blaschke B Oligodendroglial tumors: refinement of candidate regions on chromosome arm 1p and correlation of 1p/19q status with survival. Brain Pathol (2004) 14:121–30. 10.1111/j.1750-3639.2004.tb00044.x 15193024 PMC8095961

[35] Smith J Alderete B Minn Y Borell TJ Perry A Mohapatra G Localization of common deletion regions on 1p and 19q in human gliomas and their association with histological subtype. Oncogene (1999) 18:4144–52. 10.1038/sj.onc.1202759 10435596

[36] Henrich KO Schwab M Westermann F. 1p36 tumor suppression—a matter of dosage? Cancer Res (2012) 72:6079–88. 10.1158/0008-5472.CAN-12-2230 23172308

[37] Ichimura K Vogazianou AP Liu L Pearson DM Bäcklund LM Plant K 1p36 is a preferential target of chromosome 1 deletions in astrocytic tumours and homozygously deleted in a subset of glioblastomas. Oncogene (2008) 27:2097–108. 10.1038/sj.onc.1210848 17934521 PMC2650419

[38] Ball MK Kollmeyer TM Praska CE McKenna ML Giannini C Raghunathan A Frequency of false-positive FISH 1p/19q codeletion in adult diffuse astrocytic gliomas. Neuro-oncol Adv (2020) 2:vdaa109. 10.1093/noajnl/vdaa109 PMC7654379 33205043

[39] Brandner S. Molecular diagnostics of adult gliomas in neuropathological practice. Acta Med Acad (2021) 50:29–46. 10.5644/ama2006-124.324 34075762

[40] Brennan CW Verhaak RGW McKenna A Campos B Noushmehr H Salama SR The somatic genomic landscape of glioblastoma. Cell (2013) 155:462–77. 10.1016/j.cell.2013.09.034 24120142 PMC3910500

[41] Zacher A Kaulich K Stepanow S Wolter M Köhrer K Felsberg J Molecular diagnostics of gliomas using next generation sequencing of a glioma-tailored gene panel. Brain Pathol (2017) 27:146–59. 10.1111/bpa.12367 26919320 PMC8029406

[42] Albertoni M Daub DM Arden KC Viars CS Powell C Van Meir EG. Genetic instability leads to loss of both p53 alleles in a human glioblastoma. Oncogene (1998) 16:321–6. 10.1038/sj.onc.1201544 9467957

[43] Shiraishi S Tada K Nakamura H Makino K Kochi M Saya H Influence of p53 mutations on prognosis of patients with glioblastoma. Cancer (2002) 95:249–57. 10.1002/cncr.10677 12124823

[44] Bielski CM Zehir A Penson AV Donoghue MTA Chatila W Armenia J Genome doubling shapes the evolution and prognosis of advanced cancers. Nat Genet (2018) 50:1189–95. 10.1038/s41588-018-0165-1 30013179 PMC6072608

[45] Zack TI Schumacher SE Carter SL Cherniack AD Saksena G Tabak B Pan-cancer patterns of somatic copy number alteration. Nat Genet (2013) 45:1134–40. 10.1038/ng.2760 24071852 PMC3966983

[46] Boisselier B Dugay F Belaud-Rotureau M-A Coutolleau A Garcion E Menei P Whole genome duplication is an early event leading to aneuploidy in *IDH*-wild type glioblastoma. Oncotarget (2018) 9:36017–28. 10.18632/oncotarget.26330 30542515 PMC6267593

[47] Carter SL Cibulskis K Helman E McKenna A Shen H Zack T Absolute quantification of somatic DNA alterations in human cancer. Nat Biotechnol (2012) 30:413–21. 10.1038/nbt.2203 22544022 PMC4383288

[48] Quinton RJ DiDomizio A Vittoria MA Kotýnková K Ticas CJ Patel S Whole-genome doubling confers unique genetic vulnerabilities on tumour cells. Nature (2021) 590:492–7. 10.1038/s41586-020-03133-3 33505027 PMC7889737

[49] Neftel C Laffy J Filbin MG Hara T Shore ME Rahme GJ An integrative model of cellular states, plasticity, and genetics for glioblastoma. Cell (2019) 178:835–49. 10.1016/j.cell.2019.06.024 31327527 PMC6703186

[50] Wang T Li B Nelson CE Nabavi S. Comparative analysis of differential gene expression analysis tools for single-cell RNA sequencing data. BMC Bioinformatics (2019) 20:40–16. 10.1186/s12859-019-2599-6 30658573 PMC6339299

[51] Wrzeszczynski KO Frank MO Koyama T Rhrissorrakrai K Robine N Utro F Comparing sequencing assays and human-machine analyses in actionable genomics for glioblastoma. Neurol Genet (2017) 3:e164–8. 10.1212/NXG.0000000000000164 28740869 PMC5506390

[52] Gray PN Dunlop CL Elliott AM. Not all next generation sequencing diagnostics are created equal: understanding the nuances of solid tumor assay design for somatic mutation detection. Cancers (2015) 7:1313–32. 10.3390/cancers7030837 26193321 PMC4586770

[53] Gilly A Southam L Suveges D Kuchenbaecker K Moore R Melloni GEM Very low-depth whole-genome sequencing in complex trait association studies. Bioinformatics (2019) 35:2555–61. 10.1093/bioinformatics/bty1032 30576415 PMC6662288

[54] NCCN. The NCCN clinical practice guidelines in oncology (NCCN Guidelines®) for guideline central nervous system cancers version 3.2024 © national comprehensive cancer network, inc. 2024. All rights reserved. Available from: https://www.nccn.org/ (Accessed October 3, 2024).

[55] Diplas BH He X Brosnan-Cashman JA Liu H Chen LH Wang Z The genomic landscape of TERT promoter wildtype-IDH wildtype glioblastoma. Nat Commun (2018) 9:2087. 10.1038/s41467-018-04448-6 29802247 PMC5970234

[56] Yeo AT Shah R Aliazis K Pal R Xu T Zhang P Driver mutations dictate the immunologic landscape and response to checkpoint immunotherapy of glioblastoma. Cancer Immunol Res (2023) 11:629–45. 10.1158/2326-6066.cir-22-0655 36881002 PMC10155040

[57] Klein AM De Queiroz RM Venkatesh D Prives C. The roles and regulation of MDM2 and MDMX: it is not just about P53. Genes Dev (2021) 35:575–601. 10.1101/gad.347872.120 33888565 PMC8091979

[58] Pellot Ortiz KI Rechberger JS Nonnenbroich LF Daniels DJ Sarkaria JN. MDM2 inhibition in the treatment of glioblastoma: from concept to clinical investigation. Biomedicines (2023) 11:1879. 10.3390/biomedicines11071879 37509518 PMC10377337

[59] An Z Aksoy O Zheng T Fan Q-W Weiss WA. Epidermal growth factor receptor (EGFR) and EGFRvIII in glioblastoma (GBM): signaling pathways and targeted therapies. Oncogene (2018) 37:1561–75. 10.1038/s41388-017-0045-7 29321659 PMC5860944

[60] Liu X Chen X Shi L Shan Q Cao Q Yue C The third-generation EGFR inhibitor AZD9291 overcomes primary resistance by continuously blocking ERK signaling in glioblastoma. J Exp Clin Cancer Res (2019) 38:219. 10.1186/s13046-019-1235-7 31122294 PMC6533774

[61] Choi SW Jung HA Cho H-J Kim TM Park C-K Nam D-H A multicenter, phase II trial of GC1118, a novel anti-EGFR antibody, for recurrent glioblastoma patients with EGFR amplification. Cancer Med (2023) 12:15788–96. 10.1002/cam4.6213 37537946 PMC10469652

