# Supplementary material for: Diagnostic challenges in complicated case of glioblastoma
Source: Pathol Oncol Res. 2024 Oct 29;30:1611875. doi: 10.3389/pore.2024.1611875 (PMC11554483; doi:10.3389/pore.2024.1611875)
Supplement: Supplementary file 1 [file Table1.docx]

**Table S1: Aberrations detected with I-FISH.** The table listed only pathologic findings for each combination of probes. The remaining nuclei revealed normal finding. M - MetaSystems, A - Abbott Molecular, E - Empire Genomics.

| **Probe** | **Supplier** | **Localization** | **Pathologic finding**  **[% of nuclei]** | **ISCN description** | **Interpretation** |
| --- | --- | --- | --- | --- | --- |
| LSI 1p36/1q25 | A | 1p36.3/1q25.2 | 17.5 | nuc ish(MEGF6/TP73x1,ANGPTL1/ABL2x2)[35/200] | 1p36 deletion |
|  |  |  |  |  |  |
| LSI 19p13/19q13 | A | 19p13.2/19q13.3 | 11.5 | nuc ish(ZNF44/ZNF443/MAN2B1x2,GLTSCR1/GLTSCR2/CRXx1) [23/200] | 19q13 deletion |
|  |  |  | 7.5 | nuc ish(ZNF44/ZNF443/MAN2B1,GLTSCR1/GLTSCR2/CRX)x4[15/200] | tetrasomy 19 |
| XL EGFR /7cen | M | 7p11.2/7cen | 10.0 | nuc ish(EGFR,D7Z1)x3[20/200] | trisomy 7 |
|  |  |  | 15.0 | nuc ish(EGFR,D7Z1)x4[30/200] | tetrasomy 7 |
|  |  |  | 8.0 | nuc ish(EGFR,D7Z1)x6[16/200] | hexasomy 7 |
| LSI PTEN/CEP10 | A | 10q23.2q23.3/10cen | 24.5 | nuc ish(PTEN,NA)x1[49/200] | monosomy 10 |
|  |  |  |  |  |  |
| MGMT-20-OR/CEP10 | E | 10q26.3/10cen | 22.5 | nuc ish(MGMT,NA)x1[45/200] | monosomy 10 |
| XL 6q21/6q23/6cen | M | 6q21/6q23.3/6cen | 10.5 | nuc ish(D6Z1x2,SEC63x1,MYBx1)[21/200] | 6q21 and 6q23 deletion |
|  |  |  | 4.0 | nuc ish(D6Z1x3,SEC63x2,MYBx2)[8/200] | 6q21 and 6q23 deletion in triploidy |
|  |  |  | 4.0 | nuc ish(D6Z1,SEC63,MYB)x3[8/200] | trisomy 6 |
| LSI TP53/CEP17 | A | 17p13.1/17cen | 10.5 | nuc ish(TP53x1,D17Z1x2)[21/200] | *TP53* gene deletion |
|  |  |  | 9.0 | nuc ish(TP53,D17Z1)x1[18/200] | monosomy 17 |
|  |  |  | 5.0 | nuc ish(TP53x2,D17Z1x4)[10/200] | *TP53* gene deletion in tetraploidy |
|  |  |  |  |  |  |
| LSI 13 (RB1)/13q34 | A | 13q14.2/13q34 | 10.5 | nuc ish(RB1,D13S1020)x1[21/200] | monosomy 13 |
| CEP X/Y | A | Xcen/Ycen | 15.0 | nuc ish(DXZ1x1,DYZ3x2)[30/200] | supernumerary Y |
|  |  |  | 3.5 | nuc ish(DXZ1,DYZ3)x2[7/200] | tetraploidy |
|  |  |  |  |  |  |
| XL CDKN2A | M | 9p21/9cen | 7 | nuc ish(CDKN2A/CDKN2B,NA)x4[14/200] | tetrasomy 9 |
