# Supplementary material for: Diagnostic challenges in complicated case of glioblastoma
Source: Pathol Oncol Res. 2024 Oct 29;30:1611875. doi: 10.3389/pore.2024.1611875 (PMC11554483; doi:10.3389/pore.2024.1611875)

**Figure S3: Detailed genomic landscape of SVs and CNAs on chromosome 20 of a human glioblastoma sample. The** plot consists of six layers. Layers include the ideogram and magnification line, followed by CNA and B Alelle Frequency, and the two innermost layers present intrachromosomal and interchromosomal SVs. Chromosome 20 has a higher variation at the end of the chromosome. It is magnified 2x from 50 Mb to 64.4 Mb (orange). The rest of chromosome 20 is not modified (blue). Chromosome 17 is shown due to interchromosomal translocation with 20 at 0.25x zoom (green).


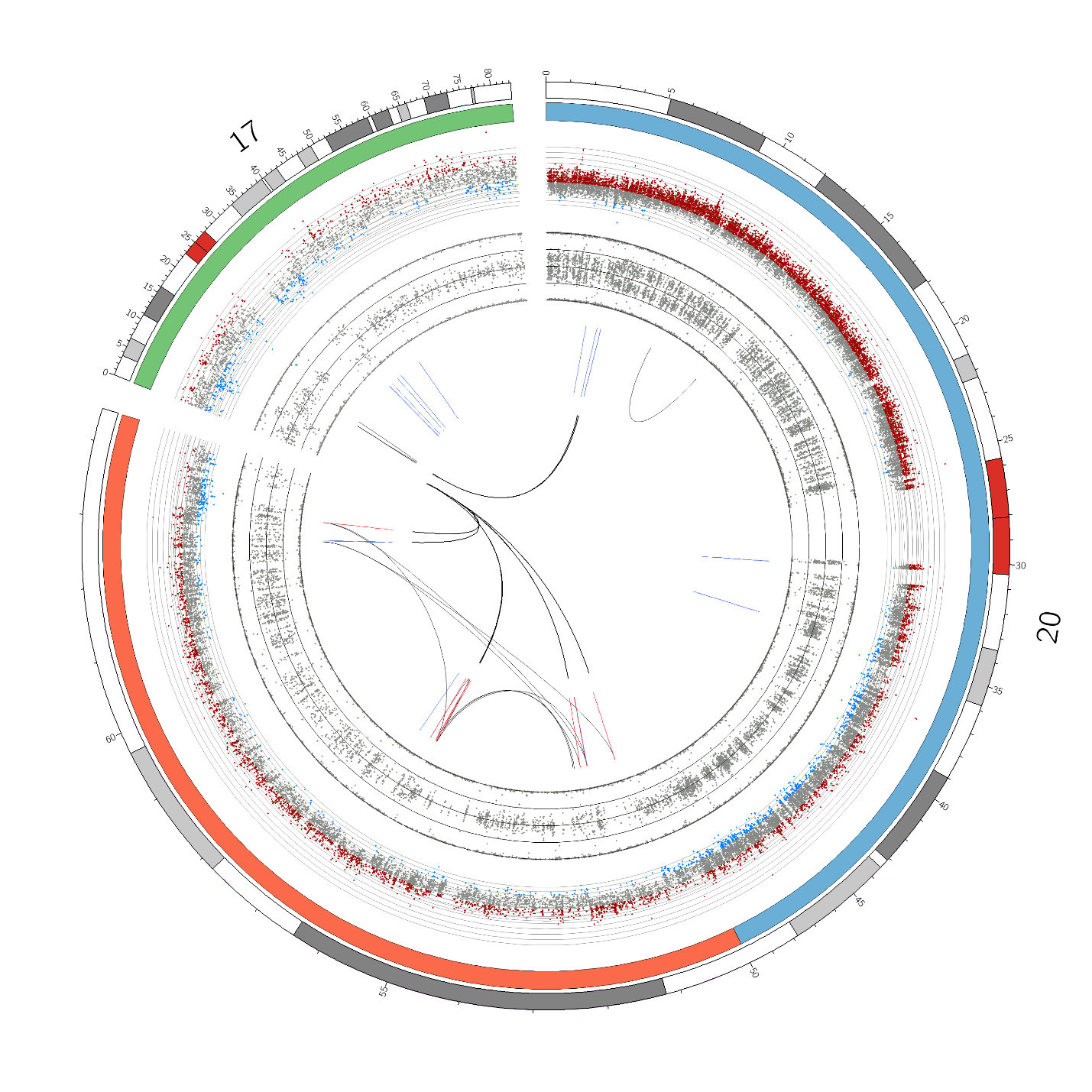

Supplement: Supplementary file 2 [file DataSheet3.docx]
