# Supplementary material for: Diagnostic challenges in complicated case of glioblastoma
Source: Pathol Oncol Res. 2024 Oct 29;30:1611875. doi: 10.3389/pore.2024.1611875 (PMC11554483; doi:10.3389/pore.2024.1611875)
Supplement: Supplementary file 3 [file Table7.docx]

**Table S7: Copy number aberration detected with WGS.**

| Chr | Start | End | Gene | Length (kb) | MedianRatio_mean | CNV _mean | BAF _mean | Status | WilcoxonRankSumTestPvalue | | TranscriptIDs | |
| --- | --- | --- | --- | --- | --- | --- | --- | --- | --- | --- | --- | --- |
| 1 | 3440000 | 11000000 | ACOT7,AJAP1,AL009183.1,AL031847.1,AL031847.2 | 7 560 | 0.7 | 1.4 | 0.737 | loss | 0 | ENST00000608083, ENST00000377842, ENST00000377845, ENST00000377855, | |  |
| 10 | 0 | 3390000 | AC026396.1,AC215217.1,ADARB2,AL139280.1,AL139280.2 | 3 390 | 0.709 | 1.41 | 0.739 | loss | 0 | ENST00000419388 | |  |
| 10 | 4780000 | 4790000 | AC018978.1,AKR1E2,AKR1C6P,AKR1E2 | 10 | 0.676 | 1.35 | 0.727 | loss | 0.000787 | ENST00000670284 | |  |
| 10 | 4940000 | 12700000 | AKR1C1,AKR1C2,AKR1C3,AKR1C4,AKR1C5P | 7 760 | 0.727 | 1.45 | 0.737 | loss | 0 | ENST00000477661, ENST00000380872, ENST00000442997, ENST00000380859, | |  |
| 10 | 12700000 | 17000000 | AC044781.1,AC069544.1,AC073367.1,ACBD7,AL139405.1 | 4 300 | 0.706 | 1.41 | 0.738 | loss | 0 | ENST00000446193 | |  |
| 10 | 17200000 | 19200000 | AC069023.1,AC069542.1,AC069542.2,AIFM1P1,AL158164.1 | 2 000 | 0.725 | 1.45 | 0.74 | loss | 0 | ENST00000442231 | |  |
| 10 | 19300000 | 22900000 | AC069549.1,AC069549.2,ADIPOR1P1,AL157398.1,AL157831.1 | 3 600 | 0.743 | 1.49 | 0.742 | loss | 0 | ENST00000451584 | |  |
| 10 | 22900000 | 26300000 | AC063961.1,AL139281.1,AL139281.2,AL139815.1,AL157385.1 | 3 400 | 0.714 | 1.43 | 0.738 | loss | 0 | ENST00000621023 | |  |
| 10 | 27000000 | 28500000 | AC022021.1,ACBD5,AL160291.1,AL355493.1,AL355493.4 | 1 500 | 0.742 | 1.48 | 0.737 | loss | 0 | ENST00000410734 | |  |
| 10 | 28600000 | 30800000 | AL158167.1,AL160060.1,AL161651.1,AL353093.1,AL353796.1 | 2 200 | 0.711 | 1.42 | 0.738 | loss | 0 | ENST00000658254 | |  |
| 10 | 32100000 | 34000000 | AK3P5,AL121748.1,AL158834.1,AL158834.2,AL161932.2 | 1 900 | 0.715 | 1.43 | 0.742 | loss | 0 | ENST00000432033 | |  |
| 10 | 34000000 | 38200000 | AL117336.1,AL117336.2,AL117337.1,AL117339.1,AL117339.2 | 4 200 | 0.722 | 1.44 | 0.742 | loss | 0 | ENST00000602435 | |  |
| 10 | 41900000 | 47200000 | AC010864.1,AC012044.1,AC068707.1,AC244230.1,AC244230.2 | 5 300 | 0.705 | 1.41 | 0.736 | loss | 0 | ENST00000609407 | |  |
| 10 | 47200000 | 50400000 | AC016397.1,AC016397.2,AC035139.1,AC035139.2,AC060234.1 | 3 200 | 0.709 | 1.42 | 0.732 | loss | 0 | ENST00000412463 | |  |
| 10 | 50400000 | 57800000 | ASAH2B,BEND3P1,CCDC58P2,CSTF2T,CTSLP4 | 7 400 | 0.745 | 1.49 | 0.74 | loss | 0 | ENST00000374007, ENST00000647317, ENST00000645350, ENST00000643851, | |  |
| 10 | 57800000 | 59400000 | BICC1,CISD1,FAM133CP,FAM13C,IPMK | 1 600 | 0.732 | 1.46 | 0.743 | loss | 0 | ENST00000373886, ENST00000476684, ENST00000263103 | |  |
| 10 | 59500000 | 61600000 | ANK3,ARL4AP1,CCDC6,CDK1,LINC00845 | 2 100 | 0.725 | 1.45 | 0.734 | loss | 0 | ENST00000280772, ENST00000373827, ENST00000373820, ENST00000502769 | |  |
| 10 | 63700000 | 64200000 | AC012558.1,AC013287.1,DBF4P1,RPL7AP50,AC013287.1 | 500 | 0.684 | 1.37 | 0.734 | loss | 6.71E-195 | ENST00000451946 | |  |
| 10 | 64200000 | 66700000 | ANXA2P3,CTNNA3,CYP2C61P,LINC01515,LINC02671 | 2 500 | 0.747 | 1.49 | 0.743 | loss | 0 | ENST00000404883 | |  |
| 10 | 66700000 | 69000000 | ACTBP14,AKR1B10P1,AL133551.1,AL139240.1,AL359844.1 | 2 300 | 0.732 | 1.46 | 0.758 | loss | 0 | ENST00000411655 | |  |
| 10 | 69200000 | 71700000 | AC016821.1,AC022532.1,AC073176.1,AC073176.2,ADAMTS14 | 2 500 | 0.692 | 1.38 | 0.729 | loss | 0 | ENST00000654329 | |  |
| 10 | 71700000 | 73000000 | ANAPC16,ASCC1,C10orf105,CDH23,CHST3 | 1 300 | 0.739 | 1.48 | 0.734 | loss | 0 | ENST00000615507, ENST00000621663, ENST00000299381, ENST00000478193 | |  |
| 10 | 73100000 | 74600000 | ANXA7,AP3M1,BMS1P4,C10orf55,CAMK2G | 1 500 | 0.745 | 1.49 | 0.74 | loss | 0 | ENST00000372921, ENST00000372919, ENST00000463788, ENST00000492380, | |  |
| 10 | 74600000 | 87700000 | ADIRF,ADK,AGAP11,AL096706.1,AL132656.1 | 13 100 | 0.711 | 1.42 | 0.736 | loss | 0 | ENST00000609170, ENST00000440490, ENST00000418273, ENST00000609111 | |  |
| 10 | 89400000 | 90700000 | IFIT5,KIF20B,LINC00865,LINC01374,LINC01375 | 1 300 | 0.725 | 1.45 | 0.747 | loss | 0 | ENST00000371795 | |  |
| 10 | 90700000 | 92700000 | ANKRD1,BTAF1,CPEB3,DDX18P6,EIF2S2P3 | 2 000 | 0.722 | 1.44 | 0.739 | loss | 0 | ENST00000371697 | |  |
| 10 | 92700000 | 92900000 | AL392103.1,AL590080.1,EXOC6 | 200 | 0.741 | 1.48 | 0.861 | loss | 2.84E-77 | ENST00000447000 | |  |
| 10 | 92900000 | 1,03E+08 | ANKRD2,ARHGAP19,ARL5AP2,ARMH3,AVPI1 | 10 100 | 0.709 | 1.42 | 0.739 | loss | 0 | ENST00000307518, ENST00000298808, ENST00000370655, ENST00000455090 | |  |
| 10 | 1,03E+08 | 1,04E+08 | CALHM1,CALHM2,CALHM3,NEURL1,PDCD11 | 1 000 | 0.676 | 1.35 | 0.729 | loss | 4.74E-217 | ENST00000329905 | |  |
| 10 | 1,04E+08 | 1,08E+08 | CFAP58,LINC01435,LINC02620,LINC02624,LINC02627 | 4 000 | 0.709 | 1.42 | 0.738 | loss | 0 | ENST00000369704 | |  |
| 10 | 1,08E+08 | 1,09E+08 | AL353740.1,LINC01435,PTGES3P5 | 1 000 | 0.719 | 1.44 | 0.73 | loss | 6.98E-179 | ENST00000366253 | |  |
| 10 | 1,09E+08 | 1,1E+08 | ADD3,AL390123.1,BTF3P15,LINC02661 | 1 000 | 0.705 | 1.41 | 0.754 | loss | 0 | ENST00000369657, ENST00000627565, ENST00000369655, ENST00000625954 | |  |
| 10 | 1,11E+08 | 1,14E+08 | AL355863.1,AL592546.2,BTBD7P2,CASP7,GPAM | 3 000 | 0.705 | 1.41 | 0.744 | loss | 0 | ENST00000424331 | |  |
| 10 | 1,14E+08 | 1,15E+08 | ATRNL1,AURKAP2,CCDC186,FAM160B1,LINC02626 | 1 000 | 0.716 | 1.43 | 0.748 | loss | 0 | ENST00000616894, ENST00000609571, ENST00000355044, ENST00000526946 | |  |
| 10 | 1,16E+08 | 1,21E+08 | BAG3,C10orf82,CACUL1,CASC2,CCDC172 | 5 000 | 0.711 | 1.42 | 0.736 | loss | 0 | ENST00000369085, ENST00000450186 | |  |
| 10 | 1,21E+08 | 1,34E+08 | ACADSB,ADAM12,ADAM8,ADGRA1,AGGF1P2 | 13 000 | 0.704 | 1.41 | 0.738 | loss | 0 | ENST00000368869, ENST00000358776, ENST00000411816, ENST00000541070 | |  |
| 11 | 9660000 | 9690000 | RN7SKP50,SWAP70 | 30 | 1.39 | 2.77 | 0.599 | gain | 1.55E-12 | ENST00000364694 | |  |
| 11 | 36300000 | 36400000 | PRR5L | 100 | 1.33 | 2.67 | 0.553 | gain | 2.36E-10 | ENST00000529034, ENST00000530639, ENST00000527172, ENST00000532121 | |  |
| 11 | 37700000 | 37800000 |  | 100 | 1.7 | 3.4 | 0.646 | gain | 1.24E-12 |  | |  |
| 11 | 73200000 | 73300000 | AP002761.4,OR8R1P | 100 | 1.52 | 3.5 | 0.927 | gain | 1.64E-12 | ENST00000565433 | |  |
| 11 | 79100000 | 79200000 | TENM4 | 100 | 1.3 | 2.6 | 0.606 | gain | 2.81E-11 | ENST00000278550, ENST00000532654, ENST00000527736, ENST00000528688 | |  |
| 12 | 40100000 | 40200000 | LINC02471,LINC02555 | 100 | 1.73 | 3.47 | 0.549 | gain | 3.21E-15 | ENST00000641941, ENST00000663154, ENST00000669755 | |  |
| 13 | 18200000 | 19700000 | ANKRD20A9P,ANKRD26P3,BNIP3P7,CASC4P1,CCNQP3 | 1 500 | 0.689 | 1.36 | 0.751 | loss | 3.13E-207 | ENST00000457997 | |  |
| 13 | 19700000 | 19800000 | LINC02371 | 100 | 0.735 | 1.47 | 0.729 | loss | 1.68E-49 | ENST00000635251, ENST00000471658, ENST00000492741, ENST00000635562 | |  |
| 13 | 19800000 | 19900000 | LINC02372 | 100 | 0.727 | 1.45 |  | loss | 2.22E-09 | ENST00000422148 | |  |
| 13 | 19900000 | 20500000 | LINC02373 | 600 | 0.727 | 1.45 | 0.746 | loss | 3.05E-223 | ENST00000624851 | |  |
| 13 | 20500000 | 22000000 | CNOT4P1,CRYL1,EEF1AKMT1,ESRRAP2,FGF9 | 1 500 | 0.731 | 1.46 | 0.736 | loss | 0 | ENST00000417432 | |  |
| 13 | 22000000 | 22600000 | AL136962.1,MTND3P1,NME1P1 | 600 | 0.723 | 1.45 | 0.741 | loss | 3.84E-206 | ENST00000657205, ENST00000631321, ENST00000611481, ENST00000659301 | |  |
| 13 | 22600000 | 22800000 | AL512484.1,DDX39AP1,FTH1P7 | 200 | 0.732 | 1.46 | 0.749 | loss | 8.72E-82 | ENST00000671380 | |  |
| 13 | 22900000 | 23000000 | AL157931.2 | 100 | 0.728 | 1.46 | 0.739 | loss | 2.09E-39 | ENST00000635091 | |  |
| 13 | 23100000 | 23700000 | HMGA1P6,LINC00327,LINC00352,LINC00362,RNU6 | 600 | 0.735 | 1.47 | 0.736 | loss | 7.96E-236 |  | |  |
| 13 | 23700000 | 23900000 | MIPEP,MTCO3P2 | 200 | 0.734 | 1.47 | 0.856 | loss | 2.18E-58 | ENST00000464194, ENST00000433710, ENST00000382172, ENST00000494139 | |  |
| 13 | 23900000 | 24700000 | ANKRD20A19P,ATP12A,C1QTNF9,C1QTNF9B,CYCSP33 | 800 | 0.732 | 1.46 | 0.743 | loss | 0.00307 | ENST00000218548, ENST00000381946 | |  |
| 13 | 24700000 | 28600000 | AMER2,ANKRD20A10P,ATP12A,ATP5F1EP2,ATP8A2 | 3 900 | 0.726 | 1.45 | 0.737 | loss | 0 | ENST00000357816, ENST00000515384 | |  |
| 13 | 28600000 | 29400000 | AL359454.1,AL359741.1,AL596092.1,CYP51A1P2,GAPDHP69 | 800 | 0.704 | 1.41 | 0.736 | loss | 0 | ENST00000620203 | |  |
| 13 | 29400000 | 29900000 | AL139188.1,LINC00297,LINC00544,LINC00572,MTUS2 | 500 | 0.734 | 1.47 | 0.73 | loss | 8.68E-186 | ENST00000619339 | |  |
| 13 | 30000000 | 30600000 | HMGB1,KATNAL1,LINC00365,LINC00384,LINC00385 | 600 | 0.727 | 1.45 | 0.739 | loss | 5.23E-218 | ENST00000341423, ENST00000405805, ENST00000339872, ENST00000399489 | |  |
| 13 | 30700000 | 33900000 | ALOX5AP,ANKRD26P4,ATP8A2P2,B3GLCT,BRCA2 | 3 200 | 0.73 | 1.46 | 0.741 | loss | 0 | ENST00000617770, ENST00000380490 | |  |
| 13 | 33900000 | 34100000 | AL160394.2,RFC3,VDAC1P12 | 200 | 0.719 | 1.44 | 0.833 | loss | 9.67E-60 | ENST00000624765 | |  |
| 13 | 34100000 | 37500000 | ALG5,ARL2BPP3,CCDC169,CCNA1,CSNK1A1L | 3 400 | 0.727 | 1.45 | 0.744 | loss | 0 | ENST00000239891, ENST00000443765, ENST00000486410, ENST00000460230 | |  |
| 13 | 38600000 | 38800000 | FREM2,LINC00366,PRDX3P3 | 200 | 0.726 | 1.45 | 0.804 | loss | 2.46E-64 | ENST00000280481 | |  |
| 13 | 38800000 | 39500000 | AL354809.1,ANKRD26P2,FREM2,LHFPL6,NHLRC3 | 700 | 0.73 | 1.46 | 0.759 | loss | 1.12E-270 | ENST00000663484, ENST00000618106, ENST00000655935, ENST00000661973 | |  |
| 13 | 39500000 | 40200000 | AL138702.1,AZU1P1,CDKN2AIPNLP3,COG6,LHFPL6 | 700 | 0.73 | 1.46 | 0.75 | loss | 2.33E-257 | ENST00000391251 | |  |
| 13 | 40200000 | 40500000 | LINC00598,RN7SKP2 | 300 | 0.73 | 1.46 | 0.75 | loss | 1.21E-87 | ENST00000615947, ENST00000638084, ENST00000654662, ENST00000637438 | |  |
| 13 | 40500000 | 40900000 | AL133318.1,AL355132.1,AL590064.1,CYCSP34,FOXO1 | 400 | 0.709 | 1.42 | 0.721 | loss | 1.96E-168 | ENST00000636651 | |  |
| 13 | 41400000 | 42400000 | AKAP11,AL157932.1,CHCHD2P11,DGKH,FABP3P2 | 1 000 | 0.745 | 1.49 | 0.74 | loss | 0 | ENST00000025301 | |  |
| 13 | 42400000 | 42700000 | LINC02341,TNFSF11 | 300 | 0.681 | 1.36 | 0.838 | loss | 2.63E-124 | ENST00000637462, ENST00000637043 | |  |
| 13 | 42700000 | 43400000 | AL138709.1,DNAJC15,ENOX1,EPSTI1,FAM216B | 700 | 0.714 | 1.43 | 0.736 | loss | 1.22E-261 | ENST00000614840 | |  |
| 13 | 43400000 | 45300000 | CCDC122,DGKZP1,ENOX1,GPALPP1,GTF2F2 | 1 900 | 0.729 | 1.46 | 0.738 | loss | 0 | ENST00000470137, ENST00000444614, ENST00000476570, ENST00000614023 | |  |
| 13 | 45300000 | 45400000 | AL138963.1,RCN1P2,SLC25A30,TPT1, | 100 | 0.742 | 1.48 | 0.87 | loss | 2.02E-23 | ENST00000420693 | |  |
| 13 | 45400000 | 45900000 | AKR1B1P4,AL139320.1,CBY2,COG3,COX4I1P2 | 500 | 0.705 | 1.41 | 0.723 | loss | 7.82E-203 | ENST00000426552 | |  |
| 13 | 46500000 | 46700000 | LRCH1 | 200 | 0.74 | 1.48 | 0.756 | loss | 2.21E-62 | ENST00000311191, ENST00000443945, ENST00000389797, ENST00000389798 | |  |
| 13 | 46700000 | 47500000 | AL359880.1,ESD,GNG5P5,HTR2A,LRCH1 | 800 | 0.724 | 1.45 | 0.75 | loss | 0 | ENST00000621879 | |  |
| 13 | 47500000 | 48200000 | AL158196.1,LINC00562,MED4,NAP1L4P3,NUDT15 | 700 | 0.703 | 1.41 | 0.74 | loss | 1.99E-229 | ENST00000616786 | |  |
| 13 | 48600000 | 48700000 | CYSLTR2,RB1 | 100 | 0.71 | 1.42 | 0.74 | loss | 5.24E-67 | ENST00000614739, ENST00000617562, ENST00000622559, ENST00000621321 | |  |
| 13 | 48700000 | 50000000 | ARL11,CAB39L,CDADC1,COX7CP1,CTAGE10P | 1 300 | 0.746 | 1.49 | 0.736 | loss | 0 | ENST00000282026, ENST00000490932 | |  |
| 13 | 50900000 | 52900000 | ALG11,ATP5PBP1,ATP7B,C13orf42,CCDC70 | 2 000 | 0.725 | 1.45 | 0.738 | loss | 0 | ENST00000523764, ENST00000521508, ENST00000649708, ENST00000616513 | |  |
| 13 | 53000000 | 55000000 | MIR1297,OLFM4,PCDH8P1,RN7SL618P,RPL13AP25 | 2 000 | 0.722 | 1.44 | 0.744 | loss | 0 | ENST00000637311 | |  |
| 13 | 58900000 | 60600000 | AL159156.1,AL359920.1,DIAPH3,HMGN2P39,LINC00434 | 1 700 | 0.728 | 1.46 | 0.74 | loss | 0 | ENST00000666115 | |  |
| 13 | 60600000 | 60900000 | AL161901.1,EIF4A1P6,LINC00378,RNA5SP31,RNY3P5 | 300 | 0.715 | 1.43 | 0.721 | loss | 3.16E-114 | ENST00000624954 | |  |
| 13 | 60900000 | 61900000 | AL592490.1,LINC00378,LINC01442,LINC02339,MIR3169 | 1 000 | 0.742 | 1.48 | 0.744 | loss | 0 | ENST00000409186 | |  |
| 13 | 62100000 | 62200000 | LINC01075 | 100 | 0.749 | 1.5 | 0.746 | loss | 8.95E-61 | ENST00000455977 | |  |
| 13 | 71900000 | 73300000 | BORA,DIS3,FABP5P1,KLF5,MZT1 | 1 400 | 0.729 | 1.46 | 0.746 | loss | 0 | ENST00000652266, ENST00000618209, ENST00000377815, ENST00000651477 | |  |
| 13 | 73300000 | 74800000 | AL138713.1,AL159972.1,AL162376.1,AL353660.1,AL355390.1 | 1 500 | 0.719 | 1.43 | 0.739 | loss | 0 | ENST00000624026 | |  |
| 13 | 74800000 | 75400000 | AL139230.1,CTAGE11P,LINC01078,RIOK3P1,RNU6 | 600 | 0.718 | 1.44 | 0.743 | loss | 1.65E-226 | ENST00000440094 | |  |
| 13 | 75400000 | 76100000 | AL137244.1,AL137782.1,COMMD6,FAM204CP,LINC00561 | 700 | 0.741 | 1.48 | 0.735 | loss | 5.64E-266 | ENST00000422931 | |  |
| 13 | 78700000 | 79200000 | CCT5P2,HSPD1P8,LINC00331,NIPA2P5, | 500 | 0.726 | 1.45 | 0.735 | loss | 1.94E-163 | ENST00000432974 | |  |
| 13 | 79200000 | 79900000 | AL136442.1,LINC01038,LINC01068,NDFIP2,RBM26 | 700 | 0.735 | 1.47 | 0.751 | loss | 8.24E-239 | ENST00000648202 | |  |
| 13 | 86200000 | 86300000 | FO624990.1 | 100 | 1.26 | 2.53 |  | gain | 0.00000736 | ENST00000636373 | |  |
| 13 | 90200000 | 92800000 | BRK1P2,FABP5P4,FAR1P1,GPC5,KRT18P27 | 2 600 | 0.744 | 1.49 | 0.744 | loss | 0 | ENST00000442875 | |  |
| 13 | 92900000 | 93000000 |  | 100 | 0.732 | 1.46 |  | loss | 1.75E-15 |  | |  |
| 13 | 93000000 | 93100000 | LINC00363 | 100 | 0.731 | 1.46 | 0.729 | loss | 1.78E-57 | ENST00000660073, ENST00000443228 | |  |
| 13 | 93100000 | 93200000 | AL354811.2 | 100 | 0.733 | 1.47 | 0.762 | loss | 1.31E-42 | ENST00000658522 | |  |
| 13 | 93200000 | 93700000 | AL160159.1,AL354811.1,GPC6,HNRNPA1P29, | 500 | 0.741 | 1.48 | 0.735 | loss | 2.15E-179 | ENST00000657026 | |  |
| 13 | 93800000 | 95000000 | BRD7P5,DCT,GPC6,GPR180,LINC00391 | 1 200 | 0.724 | 1.45 | 0.74 | loss | 0 | ENST00000299197 | |  |
| 13 | 95000000 | 95900000 | ABCC4,AL138955.1,CLDN10,DNAJC3,DZIP1 | 900 | 0.728 | 1.46 | 0.736 | loss | 0 | ENST00000645237, ENST00000643842, ENST00000643051, ENST00000646439 | |  |
| 13 | 95900000 | 99500000 | AMMECR1LP1,CALM2P4,CCR12P,CYCSP35,DOCK9 | 3 600 | 0.721 | 1.44 | 0.74 | loss | 0 | ENST00000415152 | |  |
| 13 | 99500000 | 99600000 | AL139035.2,CFL1P8,CLYBL,LINC01039,TM9SF2 | 100 | 0.744 | 1.49 | 0.875 | loss | 2.20E-28 | ENST00000654441 | |  |
| 13 | 99600000 | 1,01E+08 | AL137139.1,AL137139.2,AL137139.3,AL353697.1,AL355338.1 | 1 400 | 0.741 | 1.48 | 0.75 | loss | 0 | ENST00000364375 | |  |
| 13 | 1,01E+08 | 1,03E+08 | AL137246.1,AL137246.2,AL157769.1,AL158063.1,AL160153.1 | 2 000 | 0.735 | 1.47 | 0.746 | loss | 0 | ENST00000607072 | |  |
| 13 | 1,06E+08 | 1,08E+08 | ARGLU1,ATP5MC1P5,EFNB2,FAM155A,LIG4 | 2 000 | 0.721 | 1.44 | 0.737 | loss | 0 | ENST00000400198, ENST00000472226, ENST00000375926, ENST00000360629 | |  |
| 13 | 1,1E+08 | 1,11E+08 | AL139385.1,CARS2,COL4A2,NAXD,RAB20 | 1 000 | 0.749 | 1.5 | 0.725 | loss | 1.60E-100 | ENST00000611744 | |  |
| 13 | 1,11E+08 | 1,13E+08 | ATP11A,ATP11AUN,CUL4A,F10,F7 | 2 000 | 0.705 | 1.41 | 0.74 | loss | 0 | ENST00000487903, ENST00000375645, ENST00000375630, ENST00000419448 | |  |
| 13 | 1,13E+08 | 1,14E+08 | ADPRHL1,AL442125.1,AL442125.2,ATP4B,DCUN1D2 | 1 000 | 0.734 | 1.47 | 0.816 | loss | 3.51E-108 | ENST00000612156, ENST00000356501, ENST00000375418, ENST00000413169 | |  |
| 14 | 16100000 | 18500000 | BNIP3P6,CR383658.1,CR383658.2,RNU6, | 2 400 | 0.634 | -0.366 |  | loss | 0.127 | ENST00000549284 | |  |
| 14 | 18500000 | 21200000 | ANG,APEX1,ARHGAP42P4,ARHGAP42P5,ARHGEF40 | 2 700 | 0.733 | 1.47 | 0.744 | loss | 0 | ENST00000336811, ENST00000554073, ENST00000397990 | |  |
| 14 | 21200000 | 21400000 | HNRNPC,RPGRIP1,SUPT16H,SUPT16H, | 200 | 0.741 | 1.48 | 0.69 | loss | 5.03E-52 | ENST00000336053, ENST00000554969, ENST00000556142, ENST00000554455 | |  |
| 14 | 21400000 | 23300000 | ARL6IP1P1,BCL2L2,C14orf119,C14orf93,CDH24 | 1 900 | 0.738 | 1.48 | 0.744 | loss | 0 | ENST00000545836 | |  |
| 14 | 23300000 | 26400000 | AP1G2,BRD7P1,CARMIL3,CBLN3,CHMP4A | 3 100 | 0.711 | 1.42 | 0.734 | loss | 0 | ENST00000308724, ENST00000554892, ENST00000397120, ENST00000554554 | |  |
| 14 | 29600000 | 37100000 | AP4S1,ARHGAP5,BAZ1A,BRMS1L,CFL2 | 7 500 | 0.73 | 1.46 | 0.74 | loss | 0 | ENST00000557346, ENST00000622409, ENST00000313566, ENST00000673317 | |  |
| 14 | 37100000 | 37300000 | MIPOL1,RNU6,SLC25A21 | 200 | 0.719 | 1.44 | 0.779 | loss | 4.32E-85 | ENST00000556615, ENST00000396294, ENST00000327441, ENST00000554930 | |  |
| 14 | 37400000 | 40200000 | CLEC14A,FBXO33,FOXA1,GEMIN2,KRT8P1 | 2 800 | 0.722 | 1.44 | 0.735 | loss | 0 | ENST00000342213 | |  |
| 14 | 44300000 | 45900000 | C14orf28,DNAJC19P9,DOCK11P1,FANCM,FKBP3 | 1 600 | 0.715 | 1.43 | 0.739 | loss | 0 | ENST00000325192, ENST00000557112, ENST00000555826, ENST00000553841 | |  |
| 14 | 49000000 | 49600000 | AL110505.1,AL512361.1,RNA5SP384,RPL32P29,RPS29 | 600 | 0.696 | 1.39 | 0.753 | loss | 1.57E-229 | ENST00000557062 | |  |
| 14 | 49600000 | 51800000 | ARF6,ATL1,CDKL1,DMAC2L,DNAAF2 | 2 200 | 0.743 | 1.49 | 0.741 | loss | 0 | ENST00000298316 | |  |
| 14 | 51800000 | 52500000 | COX5AP2,GNG2,LINC02319,NID2,PTGDR | 700 | 0.725 | 1.45 | 0.736 | loss | 1.21E-237 | ENST00000554418 | |  |
| 14 | 52800000 | 59700000 | ABI1P1,ACTR10,AL049838.1,AL049873.1,AL049873.2 | 6 900 | 0.719 | 1.44 | 0.738 | loss | 0 | ENST00000603375 | |  |
| 14 | 61100000 | 61500000 | AL359220.1,PRKCH,SLC38A6,TMEM30B,PRKCH | 400 | 0.701 | 1.4 | 0.738 | loss | 6.88E-164 | ENST00000661303, ENST00000554086, ENST00000500036 | |  |
| 14 | 61800000 | 67800000 | ARG2,ATP5F1AP4,ATP6V1D,CCDC196,CHURC1 | 6 000 | 0.719 | 1.44 | 0.741 | loss | 0 | ENST00000261783, ENST00000557120, ENST00000556491, ENST00000557319 | |  |
| 14 | 68800000 | 69000000 | ACTN1,BANF1P1,BLZF2P,HMGN1P3,MAGOH3P | 200 | 0.715 | 1.43 | 0.723 | loss | 1.48E-61 | ENST00000193403, ENST00000556083, ENST00000553882, ENST00000394419 | |  |
| 14 | 69000000 | 69200000 | ACTN1,AL359317.2,AL391262.1,AL391262.2,DCAF5 | 200 | 0.741 | 1.48 | 0.799 | loss | 1.68E-97 | ENST00000193403, ENST00000394419, ENST00000438964, ENST00000376839 | |  |
| 14 | 69200000 | 71300000 | ADAM20,ADAM20P1,ADAM21,ADAM21P1,AL133445.1 | 2 100 | 0.704 | 1.41 | 0.733 | loss | 0 | ENST00000256389, ENST00000652041 | |  |
| 14 | 71700000 | 71800000 | AC004900.1,SIPA1L1 | 100 | 0.731 | 1.46 | 0.744 | loss | 3.47E-52 | ENST00000647803 | |  |
| 14 | 71800000 | 75100000 | AREL1,BBOF1,COQ6,DCAF4,DLST | 3 300 | 0.713 | 1.43 | 0.738 | loss | 0 | ENST00000554070, ENST00000557401, ENST00000356357, ENST00000555330 | |  |
| 14 | 75300000 | 76900000 | ANGEL1,BATF,CYCSP1,ERG28,ESRRB | 1 600 | 0.705 | 1.41 | 0.738 | loss | 0 | ENST00000251089, ENST00000557179, ENST00000555079, ENST00000556298 | |  |
| 14 | 77100000 | 80000000 | ADCK1,AF099810.1,AF111168.1,AF123462.1,AHSA1 | 2 900 | 0.701 | 1.4 | 0.748 | loss | 0 | ENST00000238561, ENST00000556048, ENST00000393639, ENST00000557501 | |  |
| 14 | 80000000 | 80600000 | CEP128,DIO2 | 600 | 0.733 | 1.47 | 0.753 | loss | 1.90E-221 | ENST00000554502, ENST00000556061, ENST00000281129, ENST00000555265 | |  |
| 14 | 80600000 | 86800000 | BHLHB9P1,CEP128,DYNLL1P1,DYNLL1P2,EEF1A1P2 | 6 200 | 0.743 | 1.49 | 0.74 | loss | 0 | ENST00000553845 | |  |
| 14 | 86800000 | 92500000 | ATXN3,CALM1,CAP2P1,CATSPERB,CCDC88C | 5 700 | 0.719 | 1.44 | 0.734 | loss | 0 | ENST00000642417, ENST00000644720, ENST00000564606, ENST00000642896 | |  |
| 14 | 92600000 | 1,05E+08 | ADSS1,AHNAK2,AK7,AKT1,AL049833.1 | 12 400 | 0.705 | 1.41 | 0.738 | loss | 0 | ENST00000330877, ENST00000555486, ENST00000332972, ENST00000553540 | |  |
| 14 | 1,05E+08 | 1,07E+08 | ATP5MC1P1,BRF1,BTBD6,COPDA1,CRIP1 | 2 000 | 0.694 | 1.34 | 0.774 | loss | 3.73E-163 | ENST00000448724 | |  |
| 15 | 42500000 | 42600000 | AC018362.3,HAUS2,LRRC57 | 100 | 1.44 | 2.88 | 0.706 | gain | 1.47E-09 | ENST00000650210 | |  |
| 15 | 77800000 | 78200000 | ADAMTS7P3,CIB2,COMMD4P1,CSPG4P13,IDH3A | 400 | 1.38 | 2.76 | 0.638 | gain | 1.23E-111 | ENST00000566116, ENST00000568041 | |  |
| 15 | 79900000 | 80000000 | AC015871.6,AC092701.2,BCL2A1,MTHFS,ST20 | 100 | 1.29 | 2.57 | 0.564 | gain | 1.65E-46 | ENST00000655674 | |  |
| 16 | 6770000 | 6790000 | RBFOX1 | 20 | 1.63 | 2.72 | 0.681 | gain | 9.87E-14 | ENST00000569895, ENST00000641259, ENST00000550418, ENST00000547605 | |  |
| 16 | 86100000 | 86200000 |  | 100 | 1.33 | 2.67 | 0.561 | gain | 1.61E-13 |  | |  |
| 17 | 5480000 | 7730000 | ACADVL,ACAP1,AIPL1,ALOX12,ALOX12P2 | 2 250 | 0.71 | 1.42 | 0.734 | loss | 0 | ENST00000543245, ENST00000322910, ENST00000582356, ENST00000583312, | |  |
| 17 | 27000000 | 34200000 | ADAP2,AK4P1,ALDOC,ALOX12P1,ANKRD13B | 7 200 | 0.703 | 1.41 | 0.735 | loss | 0 | ENST00000583688, ENST00000585130, ENST00000581548, ENST00000580525, ENST00000581285, ENST00000330889, ENST00000580526, ENST00000584828, ENST00000584989, ENST00000480980, ENST00000470962 | |  |
| 17 | 38900000 | 39000000 | FBXO47 | 100 | 1.71 | 3.42 | 0.629 | gain | 1.22E-09 | ENST00000378079 | |  |
| 17 | 59700000 | 59800000 | AC040904.1,MIR21,RNU6,VMP1 | 100 | 1.28 | 2.55 | 0.576 | gain | 9.78E-50 | ENST00000590850 | |  |
| 18 | 1710000 | 1730000 | AC019183.1,AP005262.2 | 20 | 1.42 | 2.85 | 0.544 | gain | 4.47E-11 | ENST00000580524 | |  |
| 18 | 27800000 | 27900000 |  | 100 | 1.68 | 3.37 | 0.585 | gain | 5.22E-10 |  | |  |
| 19 | 7060000 | 7070000 | ZNF557 | 10 | 1.71 | 3.42 | 0.585 | gain | 0.00000482 | ENST00000252840, ENST00000414706 | |  |
| 19 | 13100000 | 13200000 | AC011446.2,AC011446.3,IER2,STX10 | 100 | 1.5 | 3 | 0.567 | gain | 6.96E-07 | ENST00000586483, ENST00000592882 | |  |
| 2 | 16100000 | 16200000 | AC174048.1 | 100 | 1.75 | 3.5 | 0.57 | gain | 1.00E-12 | ENST00000603389 | |  |
| 2 | 68400000 | 68500000 | AC127383.1,FBXO48 | 100 | 1.67 | 3.35 | 0.685 | gain | 1.29E-11 | ENST00000444697 | |  |
| 2 | 1,44E+08 | 1,47E+08 | PABPC1P2,RNU7,RPL17P12,RPL6P5,TEX41 | 3 000 | 1.34 | 2.67 | 0.595 | gain | 0 | ENST00000471997 | |  |
| 2 | 1,53E+08 | 1,55E+08 | DNAJA1P2,GALNT13,KCNJ3,LINC01850,PHBP4 | 2 000 | 1.33 | 2.67 | 0.661 | gain | 0 | ENST00000421360 | |  |
| 2 | 1,55E+08 | 1,56E+08 | AC073225.1 | 1 000 | 1.42 | 2.85 | 0.665 | gain | 6.34E-214 | ENST00000662301, ENST00000665560, ENST00000666615, ENST00000670933 | |  |
| 2 | 1,56E+08 | 1,59E+08 | ACVR1,ACVR1C,BAZ2B,BTF3L4P2,CCDC148 | 3 000 | 1.32 | 2.63 | 0.656 | gain | 0 | ENST00000263640, ENST00000434821, ENST00000409283, ENST00000672582 | |  |
| 2 | 1,59E+08 | 1,6E+08 | AC009961.4,BAZ2B,CD302,LY75,MARCHF7 | 1 000 | 1.44 | 2.88 | 0.657 | gain | 2.85E-269 | ENST00000664982 | |  |
| 2 | 1,6E+08 | 1,63E+08 | AC096656.1,DPP4,EIF3EP2,FAP,GCA | 3 000 | 1.35 | 2.7 | 0.662 | gain | 0 | ENST00000623826 | |  |
| 2 | 1,94E+08 | 1,95E+08 | AC106883.1,LINC01821 | 1 000 | 1.43 | 2.86 | 0.587 | gain | 4.38E-24 | ENST00000418805 | |  |
| 20 | 60300 | 88200 | DEFB125 | 28 | 1.38 | 2.76 |  | gain | 5.22E-16 | ENST00000608838, ENST00000382410 | |  |
| 20 | 88200 | 2980000 | ANGPT4,C20orf141,C20orf202,C20orf96,CKAP2LP1 | 2 892 | 1.32 | 2.64 | 0.63 | gain | 0 | ENST00000381922 | |  |
| 20 | 4630000 | 7400000 | AL389886.1,BMP2,CASC20,CDS2,CHGB | 2 770 | 1.41 | 2.83 | 0.644 | gain | 0 | ENST00000516287 | |  |
| 20 | 7400000 | 8650000 | AL031679.1,HAO1,PHKBP1,PLCB1,RN7SL547P | 1 250 | 1.49 | 2.98 | 0.632 | gain | 0 | ENST00000400616 | |  |
| 20 | 8650000 | 8670000 | PLCB1 | 20 | 2.4 | 4.8 | 0.6 | gain | 2.07E-13 | ENST00000637919, ENST00000625874, ENST00000630495, ENST00000378641 | |  |
| 20 | 8670000 | 9070000 | AL121898.1,AL445567.1,AL445567.2,PLCB1,RNU105B | 400 | 1.36 | 2.72 | 0.643 | gain | 9.82E-204 | ENST00000616915 | |  |
| 20 | 9070000 | 10300000 | ANKEF1,HIGD1AP15,LAMP5,PAK5,PARAL1 | 1 230 | 1.32 | 2.64 | 0.615 | gain | 0 | ENST00000437504, ENST00000378392, ENST00000378380, ENST00000488991 | |  |
| 20 | 10300000 | 11500000 | AL158042.1,C20orf187,FAT1P1,JAG1,LINC01752 | 1 200 | 1.4 | 2.79 | 0.619 | gain | 0 | ENST00000605338 | |  |
| 20 | 11500000 | 14900000 | AL161938.1,BTBD3,ESF1,FLRT3,GAPDHP2 | 3 400 | 1.31 | 2.63 | 0.618 | gain | 0 | ENST00000569833 | |  |
| 20 | 14900000 | 24800000 | BANF2,BFSP1,C20orf78,CD93,CFAP61 | 9 900 | 1.31 | 2.61 | 0.619 | gain | 0 | ENST00000545418, ENST00000427254, ENST00000377805, ENST00000246090 | |  |
| 20 | 25700000 | 26100000 | BSNDP1,BSNDP2,BSNDP3,CFTRP1,FAM182A | 400 | 1.32 | 2.63 | 0.603 | gain | 1.50E-46 | ENST00000613026 | |  |
| 20 | 26100000 | 26200000 | AL121904.2,MIR663AHG | 100 |  | -2 |  | gain | 0.000661 | ENST00000657722 | |  |
| 20 | 26200000 | 31200000 | ANKRD20A21P,CDC27P3,CFTRP2,CFTRP3,DUX4L32 | 5 000 | 1.29 | 2.57 | 0.893 | gain | 2.49E-190 | ENST00000623715, ENST00000611282 | |  |
| 20 | 31200000 | 32300000 | BCL2L1,CCM2L,COX4I2,DEFB115,DEFB116 | 1 100 | 1.29 | 2.58 | 0.631 | gain | 0 | ENST00000376062, ENST00000376055, ENST00000307677, ENST00000450273 | |  |
| 20 | 32300000 | 34200000 | ASIP,ASXL1,BAK1P1,BPIFA1,BPIFA2 | 1 900 | 1.27 | 2.54 | 0.633 | gain | 0 | ENST00000568305 | |  |
| 20 | 50700000 | 50800000 | PARD6B | 100 | 1.33 | 2.65 | 0.572 | gain | 2.98E-14 | ENST00000371610, ENST00000396039 | |  |
| 20 | 50800000 | 52000000 | ATP9A,BCAS4,DPM1,KCNG1,LINC01429 | 1 200 | 1.29 | 2.58 | 0.644 | gain | 0 | ENST00000311637, ENST00000338821, ENST00000477492 | |  |
| 20 | 52000000 | 52100000 | AL109984.1,ZFP64 | 100 | 1.33 | 2.66 | 0.617 | gain | 1.26E-16 | ENST00000665639 | |  |
| 20 | 56000000 | 60900000 | ANKRD60,APCDD1L,ATP5F1E,AURKA,BMP7 | 4 900 | 1.3 | 2.6 | 0.625 | gain | 0 | ENST00000457363 | |  |
| 20 | 60900000 | 62800000 | BX640515.1,CABLES2,CDH4,COL9A3,GATA5 | 1 900 | 1.26 | 2.52 | 0.633 | gain | 0 | ENST00000617215 | |  |
| 21 | 18200000 | 18300000 | CHODL | 100 | 1.43 | 2.85 | 0.704 | gain | 1.88E-12 | ENST00000400128, ENST00000400131, ENST00000400135, ENST00000400127 | |  |
| 22 | 16800000 | 17100000 | AC007064.3,CECR7,GAB4,IGKV1OR22,IGKV2OR22 | 300 | 0.669 | 1.34 | 0.729 | loss | 1.58E-65 | ENST00000422608 | |  |
| 22 | 17100000 | 17300000 | ADA2,CECR3,FAM32BP,HDHD5,IL17RA | 200 | 0.735 | 1.47 | 0.72 | loss | 2.86E-73 | ENST00000399837, ENST00000610390, ENST00000399839, ENST00000330232 | |  |
| 22 | 17300000 | 17800000 | ATP6V1E1,BCL2L13,BID,CECR2,CECR9 | 500 | 0.722 | 1.42 | 0.75 | loss | 2.38E-176 | ENST00000253413, ENST00000399796, ENST00000399798, ENST00000473248 | |  |
| 22 | 17800000 | 18700000 | ARL2BPP10,CA15P2,FAM230A,FAM230D,FAM230J | 900 | 0.678 | 1.34 | 0.735 | loss | 8.81E-149 | ENST00000446487 | |  |
| 22 | 18900000 | 19600000 | AC007326.5,C22orf39,CA15P1,CDC45,CLDN5 | 700 | 0.7 | 1.38 | 0.742 | loss | 4.63E-235 | ENST00000640084, ENST00000638287, ENST00000623496 | |  |
| 22 | 19600000 | 21700000 | ARVCF,BCRP2,BCRP5,BCRP6,CCDC116 | 2 100 | 0.697 | 1.39 | 0.733 | loss | 0 | ENST00000263207, ENST00000495096, ENST00000401994, ENST00000406522 | |  |
| 22 | 22000000 | 24100000 | ASH2LP1,ASH2LP2,ASH2LP3,ASLP1,BCR | 2 100 | 0.684 | 1.37 | 0.74 | loss | 0 | ENST00000438037 | |  |
| 22 | 24100000 | 24700000 | ARL5AP4,BCRP1,BCRP3,CABIN1,CRIP1P4 | 600 | 0.739 | 1.48 | 0.734 | loss | 1.52E-162 | ENST00000430731 | |  |
| 22 | 24700000 | 25600000 | CRYBB2,CRYBB2P1,CRYBB3,IGLL3P,IGLVIVOR22 | 900 | 0.671 | 1.34 | 0.738 | loss | 1.28E-293 | ENST00000651629, ENST00000398215 | |  |
| 22 | 25600000 | 25800000 | AL022329.3,GRK3,MYO18B,RNA5SP494,YES1P1 | 200 | 0.73 | 1.46 | 0.729 | loss | 2.91E-70 | ENST00000624255 | |  |
| 22 | 25800000 | 27900000 | ASPHD2,CRYBA4,CRYBB1,HMGB1P10,HPS4 | 2 100 | 0.684 | 1.37 | 0.734 | loss | 0 | ENST00000215906 | |  |
| 22 | 27900000 | 28100000 | AL033538.1,AL033538.2,RN7SL757P,TTC28, | 200 | 0.696 | 1.39 | 0.76 | loss | 8.63E-67 | ENST00000615579 | |  |
| 22 | 28100000 | 29100000 | C22orf31,CCDC117,CHEK2,HSCB,KREMEN1 | 1 000 | 0.731 | 1.46 | 0.744 | loss | 0 | ENST00000216071 | |  |
| 22 | 29200000 | 29800000 | AP1B1,ASCC2,CABP7,EMID1,EWSR1 | 600 | 0.694 | 1.39 | 0.735 | loss | 1.28E-267 | ENST00000482818, ENST00000432560, ENST00000357586, ENST00000405198 | |  |
| 22 | 30000000 | 30100000 | AC003681.1,CNN2P1,HORMAD2,MIR6818,MTMR3 | 100 | 0.731 | 1.46 | 0.813 | loss | 7.72E-60 | ENST00000624945 | |  |
| 22 | 30300000 | 30400000 | CCDC157,RNF215,RNU6,SEC14L2,SF3A1 | 100 | 0.715 | 1.43 | 0.717 | loss | 5.46E-54 | ENST00000399824, ENST00000405659, ENST00000338306, ENST00000445005 | |  |
| 22 | 30500000 | 31000000 | DUSP18,EIF4HP2,GAL3ST1,MIR3200,MORC2 | 500 | 0.687 | 1.37 | 0.734 | loss | 2.79E-210 | ENST00000430175, ENST00000404885, ENST00000407308, ENST00000461301 | |  |
| 22 | 31000000 | 31300000 | INPP5J,LIMK2,MIR3928,PIK3IP1,PLA2G3 | 300 | 0.707 | 1.41 | 0.74 | loss | 3.53E-118 | ENST00000463528, ENST00000620191, ENST00000412277, ENST00000412985 | |  |
| 22 | 31300000 | 31700000 | DRG1,EIF4ENIF1,H2AZP6,LINC01521,MIR7109 | 400 | 0.707 | 1.41 | 0.83 | loss | 4.88E-179 | ENST00000331457, ENST00000416465, ENST00000433341, ENST00000486584 | |  |
| 22 | 31800000 | 32100000 | AL008719.1,AL022331.1,C22orf24,DEPDC5,LINC02558 | 300 | 0.703 | 1.41 | 0.801 | loss | 2.36E-149 | ENST00000430449 | |  |
| 22 | 32100000 | 34600000 | BPIFC,C22orf42,CPSF1P1,FBXO7,IGLCOR22 | 2 500 | 0.685 | 1.36 | 0.739 | loss | 0 | ENST00000300399, ENST00000534972, ENST00000397452, ENST00000397450 | |  |
| 22 | 34600000 | 35200000 | ISX,LINC01399,Z82196.1,Z82196.2,Z99755.1 | 600 | 0.663 | 1.33 | 0.738 | loss | 3.03E-275 | ENST00000308700, ENST00000404699 | |  |
| 22 | 35200000 | 35400000 | AL008635.1,HMGXB4,MIR3909,MIR6069,RNU7 | 200 | 0.743 | 1.49 | 0.719 | loss | 7.86E-45 | ENST00000609073 | |  |
| 22 | 35400000 | 36300000 | APOL6,HMOX1,MB,MCM5,MIR6819 | 900 | 0.704 | 1.41 | 0.728 | loss | 5.06E-273 | ENST00000409652 | |  |
| 22 | 36500000 | 36800000 | AL022313.1,AL022313.3,AL022313.4,AL049749.1,CACNG2 | 300 | 0.68 | 1.36 | 0.731 | loss | 1.21E-96 | ENST00000339367 | |  |
| 22 | 36800000 | 37000000 | CSF2RB,CSF2RBP1,IFT27,NCF4,PVALB | 200 | 0.748 | 1.5 | 0.739 | loss | 4.13E-51 | ENST00000403662, ENST00000262825, ENST00000406230, ENST00000421539 | |  |
| 22 | 37000000 | 37200000 | AL022314.1,IL2RB,KCTD17,MPST,RN7SKP214 | 200 | 0.669 | 1.34 | 0.746 | loss | 9.14E-84 | ENST00000414203 | |  |
| 22 | 37300000 | 38200000 | ANKRD54,BAIAP2L2,C22orf23,CARD10,CDC42EP1 | 900 | 0.703 | 1.41 | 0.733 | loss | 0 | ENST00000215941, ENST00000498417, ENST00000406423, ENST00000609454 | |  |
| 22 | 38200000 | 38700000 | CBY1,CSNK1E,DDX17,DMC1,FAM227A | 500 | 0.745 | 1.49 | 0.748 | loss | 3.82E-164 | ENST00000475924, ENST00000485501, ENST00000411557, ENST00000396811 | |  |
| 22 | 38700000 | 39700000 | APOBEC3A,APOBEC3B,APOBEC3C,APOBEC3D,APOBEC3F | 1 000 | 0.708 | 1.42 | 0.732 | loss | 0 | ENST00000495988, ENST00000402255, ENST00000488758, ENST00000618553 | |  |
| 22 | 39700000 | 40100000 | AL022319.1,ENTHD1,FAM83F,GRAP2,RN7SKP210 | 400 | 0.736 | 1.47 | 0.728 | loss | 3.88E-159 | ENST00000458183 | |  |
| 22 | 40100000 | 40500000 | ADSL,AL022238.1,AL022238.2,AL022238.3,AL022238.4 | 400 | 0.736 | 1.47 | 0.765 | loss | 6.45E-133 | ENST00000623632, ENST00000623063, ENST00000342312, ENST00000636714 | |  |
| 22 | 40500000 | 40700000 | COX6B1P3,MRTFA,RPL4P6 | 200 | 0.748 | 1.5 | 0.926 | loss | 2.32E-47 | ENST00000312310 | |  |
| 22 | 40700000 | 40800000 | JTBP1,SLC25A17,Z98048.1 | 100 | 0.679 | 1.36 | 0.771 | loss | 8.38E-52 | ENST00000441314 | |  |
| 22 | 40800000 | 40900000 | DNAJB7,MIR4766,SLC25A17,ST13,XPNPEP3 | 100 | 0.736 | 1.47 | 0.752 | loss | 4.13E-32 | ENST00000307221 | |  |
| 22 | 41200000 | 41400000 | CHADL,L3MBTL2,MIR6889,RANGAP1,RNU6 | 200 | 0.687 | 1.37 | 0.725 | loss | 1.09E-84 | ENST00000417999, ENST00000216241, ENST00000455425 | |  |
| 22 | 41500000 | 42900000 | A4GALT,AC254562.1,AC254562.2,AC254562.3,ACO2 | 1 400 | 0.703 | 1.41 | 0.728 | loss | 0 | ENST00000401850, ENST00000642412, ENST00000249005, ENST00000381278 | |  |
| 22 | 43000000 | 43700000 | AL022237.1,AL022476.1,BIK,BX546033.1,EFCAB6 | 700 | 0.674 | 1.35 | 0.732 | loss | 3.99E-278 | ENST00000446663 | |  |
| 22 | 43700000 | 44200000 | AL031595.1,AL031595.2,AL031595.3,AL033543.1,AL035398.1 | 500 | 0.693 | 1.39 | 0.731 | loss | 4.26E-199 | ENST00000623969 | |  |
| 22 | 44300000 | 45100000 | AL023973.1,AL079301.1,ANP32BP2,ARHGAP8,KRT18P23 | 800 | 0.684 | 1.37 | 0.736 | loss | 0 | ENST00000624619 | |  |
| 22 | 45100000 | 45400000 | AL008718.1,AL008718.2,AL008718.3,FAM118A,KIAA0930 | 300 | 0.742 | 1.48 | 0.754 | loss | 1.16E-99 | ENST00000445867 | |  |
| 22 | 45400000 | 45600000 | AL021391.1,FBLN1,LINC01589,RIBC2,RNU6 | 200 | 0.67 | 1.34 | 0.738 | loss | 6.95E-95 | ENST00000454439 | |  |
| 22 | 45600000 | 45900000 | ATXN10,MIR4762,Z84478.1,Z95331.1, | 300 | 0.731 | 1.46 | 0.777 | loss | 6.52E-90 | ENST00000381061, ENST00000252934, ENST00000640901, ENST00000498009 | |  |
| 22 | 45900000 | 46000000 | BX324167.1,BX324167.2,WNT7B | 100 | 0.624 | 1.25 | 0.737 | loss | 1.38E-61 | ENST00000451118, ENST00000650444 | |  |
| 22 | 46100000 | 46700000 | AL021392.1,AL031597.1,CDPF1,CELSR1,CERK | 600 | 0.705 | 1.41 | 0.73 | loss | 2.81E-264 | ENST00000426112 | |  |
| 22 | 46900000 | 49900000 | ALG12,BRD1,BX284656.1,BX284656.2,C22orf34 | 3 000 | 0.688 | 1.38 | 0.74 | loss | 0 | ENST00000330817, ENST00000486602, ENST00000492791 | |  |
| 22 | 50000000 | 50300000 | AL034546.1,CR559946.1,CR559946.2,DENND6B,HDAC10 | 300 | 0.704 | 1.41 | 0.749 | loss | 3.91E-137 | ENST00000623740 | |  |
| 22 | 50300000 | 50500000 | ADM2,LMF2,MIOX,NCAPH2,PPP6R2 | 200 | 0.707 | 1.41 | 0.716 | loss | 4.94E-72 | ENST00000395737, ENST00000395738 | |  |
| 3 | 4980000 | 4990000 | BHLHE40 | 10 | 1.5 | 3.1 | 0.566 | gain | 1.40E-09 | ENST00000620618, ENST00000615178, ENST00000441386, ENST00000668962 | |  |
| 3 | 21100000 | 21200000 | AC099753.1,SGO1 | 100 | 1.4 | 2.8 | 0.556 | gain | 1.37E-13 | ENST00000634947 | |  |
| 3 | 32900000 | 33000000 | CCR4 | 100 | 1.54 | 3.7 | 0.734 | gain | 9.53E-12 | ENST00000330953 | |  |
| 3 | 38100000 | 38200000 | ACAA1,MYD88,OXSR1 | 100 | 1.26 | 2.52 | 0.648 | gain | 7.29E-16 | ENST00000301810, ENST00000411549, ENST00000627515, ENST00000625927 | |  |
| 4 | 43400000 | 43500000 | LINC02383 | 100 | 1.36 | 2.71 | 0.617 | gain | 4.11E-12 | ENST00000508563 | |  |
| 5 | 4180000 | 4200000 |  | 20 | 1.61 | 3.21 | 0.703 | gain | 6.74E-12 |  | |  |
| 5 | 10300000 | 10400000 | AC012640.4,AC012640.7,MARCHF6 | 100 | 1.67 | 3.33 | 0.727 | gain | 4.52E-10 | ENST00000561606 | |  |
| 5 | 59200000 | 59500000 | AC008833.1,PDE4D | 300 | 1.28 | 2.56 | 0.592 | gain | 3.88E-134 | ENST00000611633 | |  |
| 5 | 92300000 | 92400000 | AC114316.1 | 100 | 1.75 | 3.5 | 0.532 | gain | 2.22E-11 | ENST00000507217, ENST00000502934, ENST00000654730, ENST00000661841 | |  |
| 6 | 1,63E+08 | 1,64E+08 | AL031121.1,AL031121.2,AL031121.3,AL078602.1,AL445307.1 | 1 000 | 1.33 | 2.65 | 0.718 | gain | 4.26E-157 | ENST00000406553 | |  |
| 7 | 199000 | 200000 | FAM20C | 1 | 1.54 | 3.8 |  | gain | 0.0844 | ENST00000313766, ENST00000477004 | |  |
| 7 | 200000 | 317000 | AC145676.1,AC187653.1,FAM20C | 117 | 1.59 | 3.18 | 0.646 | gain | 9.10E-61 | ENST00000514988 | |  |
| 7 | 317000 | 318000 |  | 1 | 1.48 | 2.95 |  | gain | 0.0856 |  | |  |
| 7 | 318000 | 358000 | AC188616.1 | 40 | 1.76 | 3.51 | 0.806 | gain | 3.84E-25 | ENST00000670013 | |  |
| 7 | 358000 | 365000 |  | 7 | 1.63 | 3.27 |  | gain | 0.000542 |  | |  |
| 7 | 365000 | 2290000 | ADAP1,C7orf50,COX19,CYP2W1,DNAAF5 | 1 925 | 1.59 | 3.19 | 0.608 | gain | 0 | ENST00000265846, ENST00000617043, ENST00000449296, ENST00000611167, | |  |
| 7 | 2290000 | 2500000 | AC004840.2,CHST12,EIF3B,GRIFIN,SNX8 | 210 | 1.67 | 3.34 | 0.588 | gain | 3.25E-120 | ENST00000603368 | |  |
| 7 | 2500000 | 2510000 | LFNG | 10 | 1.52 | 3.3 |  | gain | 0.00000166 | ENST00000402506 | |  |
| 7 | 2510000 | 2520000 | LFNG | 10 | 1.67 | 3.35 | 0.909 | gain | 2.16E-07 | ENST00000402506, ENST00000402045, ENST00000338732, ENST00000222725 | |  |
| 7 | 2520000 | 2540000 | BRAT1,LFNG,MIR4648 | 20 | 1.93 | 3.86 |  | gain | 0.00000467 | ENST00000340611, ENST00000493232, ENST00000467558, ENST00000469750 | |  |
| 7 | 2540000 | 6410000 | ACTB,AIMP2,AMZ1,ANKRD61,AP5Z1 | 3 870 | 1.65 | 3.3 | 0.582 | gain | 0 | ENST00000646664, ENST00000464611, ENST00000425660, ENST00000462494 | |  |
| 7 | 6420000 | 6460000 | DAGLB,KDELR2 | 40 | 1.77 | 3.54 | 0.858 | gain | 2.98E-21 | ENST00000462934, ENST00000297056, ENST00000425398, ENST00000436575, ENST00000454738, ENST00000432248, ENST00000479922, ENST00000483716 | |  |
| 7 | 6460000 | 6660000 | AC072052.1,AC079742.1,C7orf26,DAGLB,GRID2IP | 200 | 1.6 | 3.19 | 0.567 | gain | 3.02E-120 | ENST00000496556 | |  |
| 7 | 6660000 | 7050000 | ALG1L5P,CCZ1B,FAM86LP,OR7E136P,OR7E39P | 390 | 1.67 | 3.35 | 0.719 | gain | 3.39E-64 | ENST00000482043 | |  |
| 7 | 7050000 | 7070000 | AC079804.3,MIR3683 | 20 | 1.81 | 3.62 | 0.791 | gain | 2.07E-13 | ENST00000671358 | |  |
| 7 | 7070000 | 7100000 | AC011230.1,AC079804.3,AC092104.1 | 30 | 1.76 | 3.51 |  | gain | 6.14E-22 | ENST00000364348 | |  |
| 7 | 7100000 | 7670000 | AC004948.1,AC004982.1,AC004982.2,AC005532.1,AC005532.2 | 570 | 1.78 | 3.56 | 0.546 | gain | 0 | ENST00000609858 | |  |
| 7 | 7670000 | 7690000 | AC007161.3,RPA3,UMAD1 | 20 | 2.52 | 5.4 | 1 | gain | 9.49E-15 | ENST00000469183 | |  |
| 7 | 7690000 | 8070000 | CCNB2P1,GLCCI1,RNU6,RPA3,UMAD1 | 380 | 1.84 | 3.69 | 0.557 | gain | 5.73E-228 | ENST00000399411 | |  |
| 7 | 8070000 | 8780000 | AC007128.1,AC007128.2,GLCCI1,ICA1, | 710 | 1.68 | 3.37 | 0.556 | gain | 0 | ENST00000424460 | |  |
| 7 | 8780000 | 10300000 | AC004852.1,AC004852.2,AC004852.3,AC004879.1,AC004879.2 | 1 520 | 1.86 | 3.72 | 0.55 | gain | 0 | ENST00000605206 | |  |
| 7 | 10300000 | 11100000 | AC009945.1,HSPA8P8,NDUFA4,PHF14,RPL23AP52 | 800 | 1.76 | 3.51 | 0.552 | gain | 0 | ENST00000453188 | |  |
| 7 | 11100000 | 11200000 | AC004160.1,PHF14 | 100 | 2 | 4 | 0.555 | gain | 2.45E-59 | ENST00000625468, ENST00000599917 | |  |
| 7 | 11200000 | 13400000 | ARL4A,NPM1P11,RBMX2P4,RN7SKP228,SCIN | 2 200 | 1.82 | 3.63 | 0.554 | gain | 0 | ENST00000396662, ENST00000356797, ENST00000396664, ENST00000439721 | |  |
| 7 | 13400000 | 15300000 | AGMO,DGKB,EEF1A1P26,ETV1,GTF3AP5 | 1 900 | 1.84 | 3.67 | 0.557 | gain | 0 | ENST00000342526 | |  |
| 7 | 15300000 | 15800000 | AC005550.1,AC005550.2,AC006041.2,AGMO,LINC02587 | 500 | 1.97 | 3.39 | 0.551 | gain | 2.39E-293 | ENST00000438923 | |  |
| 7 | 15800000 | 20300000 | AGR2,AGR3,AHR,ANKMY2,BRWD1P3 | 4 500 | 1.78 | 3.56 | 0.563 | gain | 0 | ENST00000419304, ENST00000450569, ENST00000401412, ENST00000412973 | |  |
| 7 | 20300000 | 20500000 | AC004130.1,AC004130.2,AC099342.1,ITGB8, | 200 | 1.96 | 3.93 | 0.55 | gain | 1.14E-146 | ENST00000363883 | |  |
| 7 | 20500000 | 24100000 | AK3P3,ASS1P11,CCDC126,CDCA7L,CLK2P1 | 3 600 | 1.74 | 3.47 | 0.566 | gain | 0 | ENST00000431883 | |  |
| 7 | 24100000 | 24300000 | AC003044.1,AC004485.1,NPY,AC003044.1, | 200 | 1.71 | 3.42 | 0.809 | gain | 5.49E-124 | ENST00000662001 | |  |
| 7 | 24300000 | 26200000 | AC091705.1,C7orf31,CYCS,GSDME,HNRNPA2B1 | 1 900 | 1.72 | 3.44 | 0.559 | gain | 0 | ENST00000412197 | |  |
| 7 | 26200000 | 27100000 | AC010677.2,CBX3,HMGB3P20,KIAA0087,LINC02860 | 900 | 1.81 | 3.63 | 0.562 | gain | 0 | ENST00000650328 | |  |
| 7 | 27100000 | 27500000 | AC073150.1,EIF4HP1,EVX1,HIBADH,HNRNPA1P73 | 400 | 1.62 | 3.23 | 0.573 | gain | 2.84E-280 | ENST00000450406 | |  |
| 7 | 27500000 | 27800000 | AC005091.1,AC007130.1,HIBADH,TAX1BP1, | 300 | 1.87 | 3.73 | 0.551 | gain | 2.04E-167 | ENST00000441955 | |  |
| 7 | 27800000 | 30600000 | CHN2,CPVL,CREB5,DPY19L2P3,FKBP14 | 2 800 | 1.73 | 3.47 | 0.564 | gain | 0 | ENST00000439384, ENST00000461824, ENST00000474070, ENST00000470261 | |  |
| 7 | 30600000 | 31100000 | ADCYAP1R1,AQP1,CRHR2,GHRHR,INMT | 500 | 1.55 | 3.11 | 0.558 | gain | 9.13E-281 | ENST00000304166, ENST00000614107, ENST00000409363, ENST00000431811 | |  |
| 7 | 31100000 | 35300000 | AVL9,BBS9,BMPER,DPY19L1,DPY19L1P1 | 4 200 | 1.72 | 3.44 | 0.565 | gain | 0 | ENST00000485228, ENST00000318709, ENST00000459629, ENST00000409301 | |  |
| 7 | 35300000 | 35600000 | AC007652.1,AC007652.2,AC007652.3,AC018647.3, | 300 | 1.63 | 3.25 | 0.557 | gain | 3.19E-176 | ENST00000441150 | |  |
| 7 | 35600000 | 39100000 | AMPH,ANLN,AOAH,EEPD1,ELMO1 | 3 500 | 1.77 | 3.54 | 0.564 | gain | 0 | ENST00000356264, ENST00000325590, ENST00000441628, ENST00000460887 | |  |
| 7 | 39100000 | 43600000 | C7orf25,CDK13,CICP22,GLI3,HECW1 | 4 500 | 1.72 | 3.44 | 0.568 | gain | 0 | ENST00000350427, ENST00000447342, ENST00000431882, ENST00000438029, ENST00000425683, ENST00000432637 | |  |
| 7 | 43600000 | 43700000 | COA1,STK17A | 100 | 1.93 | 3.85 | 0.553 | gain | 2.96E-78 | ENST00000415076, ENST00000446330, ENST00000446564, ENST00000438444 | |  |
| 7 | 43700000 | 46000000 | ADCY1,AEBP1,BLVRA,CAMK2B,CCDC201 | 2 300 | 1.67 | 3.34 | 0.572 | gain | 0 | ENST00000432715, ENST00000297323, ENST00000621543, ENST00000646653, | |  |
| 7 | 46000000 | 47200000 | AC073115.1,EPS15P1,HMGN1P19,MRPL42P4,TTC4P1 | 1 200 | 1.78 | 3.57 | 0.568 | gain | 0 | ENST00000436056 | |  |
| 7 | 47200000 | 47900000 | AC087175.1,C7orf65,C7orf69,LINC00525,LINC01447 | 700 | 1.66 | 3.33 | 0.557 | gain | 0 | ENST00000656460 | |  |
| 7 | 47900000 | 49800000 | C7orf57,CDC14C,DDX43P2,GDI2P1,HUS1 | 1 900 | 1.74 | 3.48 | 0.552 | gain | 0 | ENST00000420324, ENST00000539619, ENST00000435376, ENST00000430738 | |  |
| 7 | 49800000 | 49900000 | VWC2,ZPBP | 100 | 1.67 | 3.35 |  | gain | 1.11E-09 | ENST00000340652 | |  |
| 7 | 49900000 | 50000000 | GNL2P1,VWC2,ZPBP | 100 | 1.83 | 3.66 | 0.69 | gain | 1.03E-63 | ENST00000451274 | |  |
| 7 | 50000000 | 50100000 | AC034148.1,SPATA48,ZPBP,AC020743.1,SPATA48 | 100 | 1.82 | 3.63 | 0.816 | gain | 7.85E-79 | ENST00000663206 | |  |
| 7 | 50100000 | 54400000 | CICP17,COBL,DDC,FIGNL1,GRB10 | 4 300 | 1.76 | 3.51 | 0.562 | gain | 0 | ENST00000418553 | |  |
| 7 | 54400000 | 55800000 | CALM1P2,CDC42P2,CICP11,CICP12,EGFR | 1 400 | 1.72 | 3.43 | 0.565 | gain | 0 | ENST00000452941 | |  |
| 7 | 55800000 | 56200000 | CCT6A,CHCHD2,MRPS17,NIPSNAP2,NUPR2 | 400 | 1.69 | 3.38 | 0.857 | gain | 1.20E-190 | ENST00000335503, ENST00000275603, ENST00000493855, ENST00000482776 | |  |
| 7 | 56200000 | 57400000 | AC237221.2,CCNJP1,CICP8,GUSBP10,GUSBP12 | 1 200 | 1.63 | 3.26 | 0.564 | gain | 0 | ENST00000624231 | |  |
| 7 | 57400000 | 57500000 | AC092175.1,SAPCD2P2,VN1R28P,ZNF716, | 100 | 1.85 | 3.71 | 0.546 | gain | 4.88E-38 | ENST00000605325 | |  |
| 7 | 57500000 | 57900000 | AC023141.1,AC023141.10,AC023141.11,AC023141.12,AC023141.13 | 400 | 1.75 | 3.5 | 0.559 | gain | 1.83E-39 | ENST00000419418 | |  |
| 7 | 58000000 | 62600000 | AC128676.1 | 4 600 | 1.8 | 3.59 | 0.726 | gain | 3.31E-223 | ENST00000454392 | |  |
| 7 | 62600000 | 62800000 |  | 200 | 1.78 | 3.56 | 0.557 | gain | 3.76E-145 |  | |  |
| 7 | 62800000 | 65000000 | ARAFP1,ARAFP2,ARAFP3,BNIP3P11,BNIP3P42 | 2 200 | 1.69 | 3.39 | 0.567 | gain | 0 | ENST00000422172 | |  |
| 7 | 65200000 | 67300000 | ASL,CCT6P1,CRCP,GTF2IP23,GTF2IP5 | 2 100 | 1.72 | 3.44 | 0.573 | gain | 0 | ENST00000672586, ENST00000304874, ENST00000673518, ENST00000487982 | |  |
| 7 | 67300000 | 68400000 | AC005482.1,AC006013.1,AC092637.1,AC092648.1,AC093655.1 | 1 100 | 1.58 | 3.16 | 0.575 | gain | 0 | ENST00000420758 | |  |
| 7 | 68400000 | 68500000 |  | 100 | 1.94 | 3.88 | 0.571 | gain | 1.34E-41 |  | |  |
| 7 | 68500000 | 69200000 | AC004910.1,AC069280.1,AC069280.2,AC092100.1,AC104688.1 | 700 | 1.6 | 3.2 | 0.578 | gain | 0 | ENST00000433493 | |  |
| 7 | 69200000 | 69400000 | AC092100.1,MTCO1P25,MTCO2P25,RNU6,AC092100.1 | 200 | 1.58 | 3.15 | 0.743 | gain | 2.82E-106 | ENST00000670444, ENST00000435148, ENST00000426356, ENST00000421513 | |  |
| 7 | 69400000 | 69600000 | AC004966.1,AC092100.1 | 200 | 1.62 | 3.25 | 0.568 | gain | 1.24E-76 | ENST00000363987 | |  |
| 7 | 69600000 | 70300000 | AUTS2,CT66 | 700 | 1.71 | 3.42 | 0.552 | gain | 0 | ENST00000644939, ENST00000342771, ENST00000406775, ENST00000403018 | |  |
| 7 | 70300000 | 72000000 | AC005011.1,AC073873.1,AUTS2,CALN1,GALNT17 | 1 700 | 1.61 | 3.22 | 0.571 | gain | 0 | ENST00000455206 | |  |
| 7 | 72000000 | 72500000 | CALN1 | 500 | 1.67 | 3.34 | 0.751 | gain | 4.00E-42 | ENST00000329008, ENST00000395275, ENST00000395276, ENST00000431984 | |  |
| 7 | 72500000 | 73600000 | BAZ1B,BCL7B,FKBP6,FZD9,GTF2IP4 | 1 100 | 1.67 | 3.35 | 0.605 | gain | 1.59E-200 | ENST00000339594, ENST00000404251, ENST00000466844 | |  |
| 7 | 73600000 | 74200000 | ABHD11,AC005089.1,AC099398.1,BUD23,CLDN3 | 600 | 1.62 | 3.24 | 0.614 | gain | 0 | ENST00000427153, ENST00000641969, ENST00000497897, ENST00000437775 | |  |
| 7 | 74200000 | 74300000 | AC005081.1,CLIP2,LAT2,RFC2,CLIP2 | 100 | 1.76 | 3.52 | 0.572 | gain | 9.29E-49 | ENST00000661689 | |  |
| 7 | 74300000 | 74400000 | CLIP2 | 100 | 1.64 | 3.29 | 0.802 | gain | 1.90E-35 | ENST00000223398, ENST00000361545 | |  |
| 7 | 74400000 | 76300000 | CASTOR2,CCL24,CCL26,CLIP2,GTF2I | 1 900 | 1.63 | 3.26 | 0.59 | gain | 0 | ENST00000616305, ENST00000622472 | |  |
| 7 | 76300000 | 79000000 | APTR,CCDC146,DTX2,DTX2P1,FAM185BP | 2 700 | 1.75 | 3.51 | 0.567 | gain | 0 | ENST00000659053, ENST00000440088, ENST00000665948, ENST00000430801 | |  |
| 7 | 79000000 | 79300000 | AC006355.1,AC006355.2,MAGI2,RNU6,MAGI2 | 300 | 1.72 | 3.44 | 0.684 | gain | 2.43E-178 | ENST00000440982 | |  |
| 7 | 79300000 | 91600000 | ADAM22,CACNA2D1,CD36,CDK14,CFAP69 | 12 300 | 1.81 | 3.62 | 0.569 | gain | 0 | ENST00000398204, ENST00000439864, ENST00000412441, ENST00000398201 | |  |
| 7 | 91600000 | 92800000 | AKAP9,ANKIB1,CDK6,CYP51A1,ERVW | 1 200 | 1.77 | 3.55 | 0.695 | gain | 0 | ENST00000356239, ENST00000493453, ENST00000619023, ENST00000394564 | |  |
| 7 | 92800000 | 98800000 | AP1S2P1,ARF1P1,ASB4,ASNS,ATP5PBP2 | 6 000 | 1.74 | 3.48 | 0.569 | gain | 0 | ENST00000457344 | |  |
| 7 | 98800000 | 99200000 | AC004893.3,MIR3609,SMURF1,TMEM130,TRRAP | 400 | 1.59 | 3.17 | 0.57 | gain | 1.92E-186 | ENST00000663185 | |  |
| 7 | 99200000 | 99800000 | ARPC1A,ARPC1B,ATP5MF,BUD31,CPSF4 | 600 | 1.64 | 3.29 | 0.673 | gain | 0 | ENST00000262942, ENST00000432786, ENST00000471960, ENST00000463009 | |  |
| 7 | 99800000 | 1E+08 | AP4M1,AZGP1,AZGP1P1,CASTOR3,CNPY4 | 200 | 1.68 | 3.36 | 0.563 | gain | 6.78E-239 | ENST00000394061, ENST00000438383, ENST00000445208, ENST00000429084 | |  |
| 7 | 1E+08 | 1,01E+08 | ACTL6B,AGFG2,C7orf61,EPHB4,EPO | 1 000 | 1.67 | 3.34 | 0.765 | gain | 1.02E-231 | ENST00000160382, ENST00000487125, ENST00000485601, ENST00000487225 | |  |
| 7 | 1,01E+08 | 1,02E+08 | AC004965.1,AZGP1P2,COL26A1,EMSLR,IFT22 | 1 000 | 1.55 | 3.9 | 0.582 | gain | 2.66E-222 | ENST00000413033 | |  |
| 7 | 1,02E+08 | 1,05E+08 | ALKBH4,ARMC10,CRYZP1,CUX1,DNAJC2 | 3 000 | 1.7 | 3.4 | 0.56 | gain | 0 | ENST00000292566, ENST00000490528, ENST00000498283 | |  |
| 7 | 1,05E+08 | 1,06E+08 | ATXN7L1,CDHR3,DCAF13P1,EFCAB10,KMT2E | 1 000 | 1.71 | 3.43 | 0.566 | gain | 0 | ENST00000419735, ENST00000477775, ENST00000484475, ENST00000474433 | |  |
| 7 | 1,06E+08 | 1,07E+08 | AC007032.1,CCDC71L,LARP1BP2,LINC02577,NAMPT | 1 000 | 1.77 | 3.53 | 0.554 | gain | 0 | ENST00000609281 | |  |
| 7 | 1,07E+08 | 1,08E+08 | AC005046.2,BANF1P5,BCAP29,CBLL1,COG5 | 1 000 | 1.76 | 3.52 | 0.568 | gain | 0 | ENST00000608515 | |  |
| 7 | 1,08E+08 | 1,13E+08 | BUB3P1,C7orf66,DNAJB9,DOCK4,EIF3IP1 | 5 000 | 1.77 | 3.54 | 0.56 | gain | 0 | ENST00000458451 | |  |
| 7 | 1,13E+08 | 1,26E+08 | ANKRD7,ASB15,ASZ1,BMT2,C7orf77 | 13 000 | 1.8 | 3.6 | 0.572 | gain | 0 | ENST00000433239, ENST00000477532, ENST00000265224, ENST00000486422 | |  |
| 7 | 1,26E+08 | 1,28E+08 | ARF5,FSCN3,GCC1,GRM8,LRRC4 | 2 000 | 1.71 | 3.41 | 0.562 | gain | 0 | ENST00000463733, ENST00000415666, ENST00000467281, ENST00000000233 | |  |
| 7 | 1,28E+08 | 1,31E+08 | AHCYL2,ATP6V1F,ATP6V1FNB,AUXG01000058.1,CALU | 3 000 | 1.71 | 3.42 | 0.586 | gain | 0 | ENST00000325006, ENST00000446544, ENST00000461161, ENST00000460109 | |  |
| 7 | 1,31E+08 | 1,33E+08 | EEF1B2P6,NDUFB9P2,PLXNA4,PODXL,AC007790.1 | 2 000 | 1.59 | 3.18 | 0.573 | gain | 0 | ENST00000445713 | |  |
| 7 | 1,33E+08 | 1,34E+08 | AC083875.1,CHCHD3,COX5BP3,EXOC4,LRGUK | 1 000 | 1.71 | 3.42 | 0.571 | gain | 0 | ENST00000419509 | |  |
| 7 | 1,34E+08 | 1,35E+08 | AKR1B1,AKR1B10,AKR1B15,BPGM,CALD1 | 1 000 | 1.65 | 3.3 | 0.552 | gain | 3.92E-245 | ENST00000285930, ENST00000434222, ENST00000465351, ENST00000467251 | |  |
| 7 | 1,35E+08 | 1,37E+08 | AGBL3,CALD1,CHRM2,CNOT4,CYREN | 2 000 | 1.77 | 3.53 | 0.565 | gain | 0 | ENST00000436302, ENST00000275763, ENST00000435976, ENST00000455283 | |  |
| 7 | 1,37E+08 | 1,4E+08 | AKR1D1,ATP6V0A4,CLEC2L,CREB3L2,DGKI | 3 000 | 1.7 | 3.4 | 0.583 | gain | 0 | ENST00000468877, ENST00000432161, ENST00000242375, ENST00000411726 | |  |
| 7 | 1,4E+08 | 1,42E+08 | ADCK2,AGK,BRAF,CCT4P1,DENND11 | 2 000 | 1.71 | 3.41 | 0.573 | gain | 0 | ENST00000072869, ENST00000476491, ENST00000483369, ENST00000498423 | |  |
| 7 | 1,42E+08 | 1,47E+08 | ARHGEF34P,ARHGEF35,ARHGEF5,CASP2,CLCN1 | 5 000 | 1.78 | 3.57 | 0.567 | gain | 0 | ENST00000483587 | |  |
| 7 | 1,47E+08 | 1,49E+08 | C7orf33,CNTNAP2,CUL1,EZH2,MIR548F4 | 2 000 | 1.72 | 3.43 | 0.551 | gain | 0 | ENST00000307003 | |  |
| 7 | 1,49E+08 | 1,53E+08 | ACTR3C,AGAP3,ALDH7A1P3,AOC1,ASB10 | 4 000 | 1.7 | 3.4 | 0.582 | gain | 0 | ENST00000478393, ENST00000613959, ENST00000252071, ENST00000539352 | |  |
| 7 | 1,54E+08 | 1,56E+08 | AF093117.1,BLACE,CNPY1,DPP6,EN2 | 2 000 | 1.67 | 3.33 | 0.578 | gain | 0 | ENST00000384333 | |  |
| 7 | 1,58E+08 | 1,59E+08 | AC019084.1,AC078942.1,ESYT2,LINC01022,MIR5707 | 1 000 | 1.67 | 3.35 | 0.661 | gain | 1.38E-249 | ENST00000457337 | |  |
| 8 | 0 | 4720000 | ARHGEF10,CLN8,CSMD1,DLGAP2,ERICH1 | 4 720 | 0.723 | 1.44 | 0.74 | loss | 0 | ENST00000349830, ENST00000520359, ENST00000518288, ENST00000398564 | |  |
| 8 | 4720000 | 4770000 | CSMD1 | 50 | 0.729 | 1.46 | 0.908 | loss | 6.47E-17 | ENST00000635120, ENST00000400186, ENST00000602723, ENST00000520002 | |  |
| 8 | 4770000 | 4790000 | CSMD1,PAICSP4 | 20 | 0.711 | 1.42 |  | loss | 7.51E-13 | ENST00000635120, ENST00000400186, ENST00000602723, ENST00000520002 | |  |
| 8 | 4790000 | 6100000 | AC091193.1,CSMD1,RN7SKP159,RN7SL318P,RPL23AP54 | 1 310 | 0.735 | 1.47 | 0.741 | loss | 0 | ENST00000604640 | |  |
| 8 | 6120000 | 15500000 | AGPAT5,ALG1L11P,ALG1L12P,ALG1L13P,ANGPT2 | 9 380 | 0.728 | 1.46 | 0.738 | loss | 0 | ENST00000285518, ENST00000523234, ENST00000518327, ENST00000523586 | |  |
| 8 | 15500000 | 16100000 | AC018437.2,AC018437.3,AC100850.1,MSR1,PPM1AP1 | 600 | 0.717 | 1.42 | 0.739 | loss | 7.03E-237 | ENST00000517369 | |  |
| 8 | 16500000 | 17800000 | AP006248.6,CNOT7,FGF20,MICU3,MIR548V | 1 300 | 0.736 | 1.47 | 0.745 | loss | 0 | ENST00000660331 | |  |
| 8 | 17900000 | 20200000 | ASAH1,CSGALNACT1,FGL1,INTS10,LPL | 2 300 | 0.733 | 1.47 | 0.742 | loss | 0 | ENST00000637790, ENST00000262097, ENST00000636009, ENST00000520781 | |  |
| 8 | 20200000 | 22400000 | ATP6V1B2,BMP1,DMTN,DOK2,FAM160B2 | 2 200 | 0.696 | 1.39 | 0.739 | loss | 0 | ENST00000276390, ENST00000523482, ENST00000521442 | |  |
| 8 | 22400000 | 22900000 | BIN3,C8orf58,CCAR2,EGR3,PDLIM2 | 500 | 0.733 | 1.47 | 0.742 | loss | 2.04E-157 | ENST00000276416, ENST00000520489, ENST00000519513, ENST00000399977 | |  |
| 8 | 22900000 | 23100000 | AC107959.5,PEBP4,RHOBTB2,RN7SL303P,TNFRSF10B | 200 | 0.726 | 1.45 | 0.71 | loss | 2.96E-92 | ENST00000520607 | |  |
| 8 | 23100000 | 23700000 | AC107959.5,CHMP7,ENTPD4,LOXL2,R3HCC1 | 600 | 0.738 | 1.48 | 0.747 | loss | 1.95E-187 | ENST00000520607 | |  |
| 8 | 23700000 | 28400000 | ADAM28,ADAM7,ADAMDEC1,ADRA1A,AF106564.1 | 4 700 | 0.714 | 1.43 | 0.737 | loss | 0 | ENST00000523379, ENST00000520448, ENST00000265769, ENST00000437154 | |  |
| 8 | 28400000 | 29200000 | EXTL3,FBXO16,FZD3,HMBOX1,HMGB1P23 | 800 | 0.754 | 1.5 | 0.759 | loss | 6.62E-293 | ENST00000522725, ENST00000523149, ENST00000519288, ENST00000520940 | |  |
| 8 | 29200000 | 30000000 | DUSP4,KIF13B,LINC00589,LINC02099,LINC02209 | 800 | 0.74 | 1.48 | 0.727 | loss | 8.87E-281 | ENST00000240100, ENST00000240101 | |  |
| 8 | 30200000 | 30600000 | DCTN6,GTF2E2,HSPA8P11,LEPROTL1,PPIAP84 | 400 | 0.689 | 1.38 | 0.766 | loss | 2.28E-167 | ENST00000518096, ENST00000248151 | |  |
| 8 | 30600000 | 31100000 | GSR,GTF2E2,PPP2CB,PURG,RNU5A | 500 | 0.743 | 1.49 | 0.742 | loss | 1.78E-188 | ENST00000221130, ENST00000643653, ENST00000643525, ENST00000537535 | |  |
| 8 | 31200000 | 31600000 | AC068672.1,AC068672.2,AC068672.3,AC090816.1,KCTD9P6 | 400 | 0.699 | 1.4 | 0.755 | loss | 2.52E-152 | ENST00000520391 | |  |
| 8 | 31600000 | 33300000 | AC104027.1,MTND1P6,MTND2P32,NRG1,RANP9 | 1 700 | 0.719 | 1.44 | 0.742 | loss | 0 | ENST00000521887 | |  |
| 8 | 33300000 | 34400000 | BUD31P1,DUSP26,FUT10,MAK16,RN7SL457P | 1 100 | 0.702 | 1.4 | 0.747 | loss | 0 | ENST00000521828 | |  |
| 8 | 34400000 | 35200000 | AC087343.1,AC099685.1,LINC01288 | 800 | 0.716 | 1.43 | 0.753 | loss | 0 | ENST00000477341 | |  |
| 8 | 34400000 | 35200000 | ADGRA2,ADRB3,AP006245.1,AP006245.2,BRF2 | 800 | 0.703 | 1.41 | 0.742 | loss | 0 | ENST00000428068, ENST00000412232, ENST00000315215 | |  |
| 8 | 38100000 | 43200000 | ADAM18,ADAM2,ADAM32,ADAM3A,ADAM5 | 5 100 | 0.725 | 1.45 | 0.738 | loss | 0 | ENST00000520001, ENST00000265707, ENST00000379866, ENST00000520772 | |  |
| 8 | 43200000 | 43900000 | AC139365.2,AFG3L2P1,CYP4F44P,POTEA,RNU6 | 700 | 0.739 | 1.48 | 0.744 | loss | 3.68E-229 | ENST00000517972 | |  |
| 8 | 43900000 | 47300000 | ASNSP1,ASNSP4,ATP6V1G1P2,HSPA8P13,IGLV8OR8 | 3 400 | 0.69 | 1.23 | 0.764 | loss | 0 | ENST00000518311, ENST00000641079, ENST00000641977, ENST00000641735 | |  |
| 8 | 47700000 | 49100000 | AC105029.1,EFCAB1,LINC02599,LINC02847,MCM4 | 1 400 | 0.704 | 1.41 | 0.738 | loss | 0 | ENST00000520250 | |  |
| 8 | 49100000 | 49200000 | AC044893.1,PPDPFL | 100 | 0.749 | 1.5 | 0.749 | loss | 1.55E-48 | ENST00000657689, ENST00000667939, ENST00000662394, ENST00000662753 | |  |
| 8 | 49300000 | 51600000 | AC113145.1,BRIX1P1,CYCSP22,PSAT1P1,PXDNL | 2 300 | 0.749 | 1.5 | 0.742 | loss | 0 | ENST00000522408 | |  |
| 8 | 51600000 | 51900000 | AC090186.1,BTF3P1,PCMTD1,PXDNL, | 300 | 0.738 | 1.48 | 0.828 | loss | 1.39E-106 | ENST00000605991 | |  |
| 8 | 52000000 | 55900000 | ALKAL1,ATP6V1H,LYPLA1,MAPK6P1,MRPL15 | 3 900 | 0.716 | 1.43 | 0.743 | loss | 0 | ENST00000358543, ENST00000523939 | |  |
| 8 | 56000000 | 56500000 | AC107952.2,CERNA3,CHCHD7,LYN,MOS | 500 | 0.722 | 1.44 | 0.727 | loss | 2.80E-172 | ENST00000606048 | |  |
| 8 | 56500000 | 58900000 | AC104350.1,CYP7A1,FAM110B,IMPAD1,LINC00588 | 2 400 | 0.747 | 1.49 | 0.741 | loss | 0 | ENST00000518750 | |  |
| 8 | 60100000 | 60200000 | AC021393.1,SLC2A13P1 | 100 | 0.698 | 1.4 | 0.931 | loss | 2.14E-09 | ENST00000655977, ENST00000665744 | |  |
| 8 | 60200000 | 62900000 | ASPH,C1GALT1P3,CA8,CHD7,CLVS1 | 2 700 | 0.737 | 1.47 | 0.739 | loss | 0 | ENST00000379454, ENST00000541428, ENST00000521909, ENST00000524173 | |  |
| 8 | 62900000 | 67400000 | ADHFE1,ARFGEF1,ARMC1,BHLHE22,COPS5 | 4 500 | 0.734 | 1.47 | 0.745 | loss | 0 | ENST00000415254, ENST00000424777, ENST00000276576, ENST00000396621 | |  |
| 8 | 67400000 | 69700000 | C8orf34,CPA6,LINC01592,LINC01603,NACAP10 | 2 300 | 0.717 | 1.43 | 0.739 | loss | 0 | ENST00000660308, ENST00000653063, ENST00000656279, ENST00000653785 | |  |
| 8 | 77800000 | 78800000 | AC100854.1,IL7,PKIA,RNU6,THAP12P7 | 1 000 | 0.75 | 1.5 | 0.79 | loss | 0 | ENST00000520351 | |  |
| 8 | 78800000 | 85300000 | ACTBP6,CA13,CHMP4C,CKS1BP7,E2F5 | 6 500 | 0.75 | 1.5 | 0.745 | loss | 0 | ENST00000520036 | |  |
| 8 | 85300000 | 86100000 | AC232323.1,ATP6V0D2,CA1,CA2,CA3 | 800 | 0.746 | 1.49 | 0.745 | loss | 3.67E-241 | ENST00000540724 | |  |
| 8 | 86200000 | 86800000 | CNGB3,CPNE3,GOLGA2P1,MIOXP1,NTAN1P2 | 600 | 0.72 | 1.41 | 0.757 | loss | 2.25E-237 | ENST00000517327, ENST00000320005, ENST00000519777 | |  |
| 8 | 86800000 | 87200000 | AC090572.2,CNBD1 | 400 | 0.736 | 1.47 | 0.765 | loss | 3.56E-146 | ENST00000520649 | |  |
| 8 | 87700000 | 89000000 | AF121898.1,DCAF4L2,MMP16,RNA5SP272,SOX5P1 | 1 300 | 0.749 | 1.5 | 0.746 | loss | 0 | ENST00000440763, ENST00000657849, ENST00000517711, ENST00000518494 | |  |
| 8 | 89100000 | 89800000 | AF117829.1,KRT8P4,RIPK2,RPSAP74, | 700 | 0.736 | 1.47 | 0.763 | loss | 5.47E-208 | ENST00000661105, ENST00000658265, ENST00000519655, ENST00000656898 | |  |
| 8 | 89900000 | 93100000 | AF181450.1,C8orf88,CALB1,DECR1,IRF5P1 | 3 200 | 0.748 | 1.5 | 0.748 | loss | 0 | ENST00000522980 | |  |
| 8 | 93100000 | 94700000 | C8orf87,CDH17,ESRP1,FAM92A,FSBP | 1 600 | 0.719 | 1.44 | 0.745 | loss | 0 | ENST00000521906, ENST00000522116, ENST00000625010 | |  |
| 8 | 94700000 | 95900000 | AP003692.1,C8orf37,CCNE2,DPY19L4,INTS8 | 1 200 | 0.731 | 1.46 | 0.738 | loss | 0 | ENST00000521706, ENST00000510185 | |  |
| 8 | 95900000 | 96600000 | GDF6,MTERF3,PTDSS1,RNU6,SDC2 | 700 | 0.709 | 1.42 | 0.738 | loss | 5.87E-293 | ENST00000287020, ENST00000621429, ENST00000620978 | |  |
| 8 | 97100000 | 98900000 | AP003467.2,CPQ,ERICH5,KCNS2,LAPTM4B | 1 800 | 0.705 | 1.41 | 0.736 | loss | 0 | ENST00000520175 | |  |
| 8 | 99500000 | 1,02E+08 | ANKRD46,AP000424.1,AP000424.2,AP000426.1,AP001205.1 | 2 500 | 0.72 | 1.44 | 0.737 | loss | 0 | ENST00000520552, ENST00000335659, ENST00000519597, ENST00000520311 | |  |
| 8 | 1,02E+08 | 1,05E+08 | ATP6V1C1,AZIN1,BAALC,CTHRC1,DCAF13 | 3 000 | 0.74 | 1.48 | 0.739 | loss | 0 | ENST00000518857, ENST00000395862, ENST00000518738, ENST00000521514 | |  |
| 8 | 1,05E+08 | 1,08E+08 | AP003789.1,HMGB1P46,NRBF2P4,OXR1,PGAM1P13 | 3 000 | 0.747 | 1.49 | 0.741 | loss | 0 | ENST00000617737 | |  |
| 8 | 1,08E+08 | 1,1E+08 | AC104248.1,EBAG9,EIF3E,EMC2,ENY2 | 2 000 | 0.742 | 1.48 | 0.744 | loss | 0 | ENST00000661381, ENST00000522244, ENST00000524134, ENST00000656015 | |  |
| 8 | 1,16E+08 | 1,2E+08 | AP005717.2,CCN3,COL14A1,COLEC10,CYCSP23 | 4 000 | 0.717 | 1.43 | 0.736 | loss | 0 | ENST00000665858 | |  |
| 8 | 1,2E+08 | 1,25E+08 | ANXA13,ARF1P3,ATAD2,C8orf76,CDK5P1 | 5 000 | 0.723 | 1.44 | 0.738 | loss | 0 | ENST00000419625, ENST00000262219, ENST00000520519, ENST00000523822 | |  |
| 8 | 1,25E+08 | 1,27E+08 | AC091114.1,KNOP1P5,LINC00861,LRATD2,PCAT1 | 2 000 | 0.718 | 1.44 | 0.739 | loss | 0 | ENST00000522815, ENST00000521991 | |  |
| 8 | 1,27E+08 | 1,36E+08 | ASAP1,CASC11,CASC19,CASC8,CCAT2 | 9 000 | 0.722 | 1.44 | 0.74 | loss | 0 | ENST00000524124, ENST00000518721, ENST00000357668, ENST00000521075 | |  |
| 8 | 1,36E+08 | 1,4E+08 | AC110053.1,C8orf17,COL22A1,FAM135B,KCNK9 | 4 000 | 0.705 | 1.41 | 0.735 | loss | 0 | ENST00000523156 | |  |
| 8 | 1,4E+08 | 1,41E+08 | AGO2,CHRAC1,DENND3,ERICD,MIR151A | 1 000 | 0.744 | 1.49 | 0.727 | loss | 2.93E-293 | ENST00000220592, ENST00000519980, ENST00000523609, ENST00000520628 | |  |
| 8 | 1,41E+08 | 1,42E+08 | AC138647.2,GPR20,HNRNPA1P38,LINC01300,MIR1302 | 1 000 | 0.685 | 1.37 | 0.723 | loss | 2.01E-210 | ENST00000667112 | |  |
| 8 | 1,42E+08 | 1,45E+08 | ADCK5,ADGRB1,AF186192.1,AF186192.2,AF186192.3 | 3 000 | 0.69 | 1.38 | 0.736 | loss | 0 | ENST00000534714, ENST00000526231, ENST00000533715, ENST00000529654 | |  |
| 9 | 1,01E+08 | 1,02E+08 | AL353621.1,PGAP4 | 1 000 | 1.47 | 2.94 | 0.549 | gain | 8.65E-13 | ENST00000418219 | |  |
| X | 41400000 | 41500000 | NYX | 100 | 0.813 | 1.63 | 0.898 | gain | 0.000019 | ENST00000342595, ENST00000378220 | |  |
| X | 50200000 | 50300000 | CCNB3 | 100 | 0.976 | 1.95 |  | gain | 0.678 | ENST00000493507, ENST00000491907, ENST00000376042, ENST00000376038 | |  |
| X | 53700000 | 53800000 | BX323845.1 | 100 | 0.896 | 1.79 | 1 | gain | 0.00842 | ENST00000432417 | |  |
| X | 85700000 | 85800000 |  | 100 | 1.3 | 2.5 | 0.5 | gain | 0.353 |  | |  |
| X | 1,21E+08 | 1,22E+08 | , | 1 000 | 0.754 | 1.51 |  | gain | 4.41E-07 |  | |  |
| Y | 0 | 2890000 | AC006040.1,HSFY3P,RNASEH2CP1,RNU6,RPS4Y1 | 2 890 | 0.808 | -0.192 |  | gain | 1.47E-24 | ENST00000651710 | |  |
| Y | 2890000 | 2900000 | RPS4Y1 | 10 | 0.834 | 1.67 |  | gain | 0.329 | ENST00000515575 | |  |
| Y | 2900000 | 6830000 | AGPAT5P1,DLGAP5P1,EEF1A1P41,EIF4A1P2,FAM197Y9 | 3 930 | 0.846 | 1.69 | 0.827 | gain | 1.22E-40 | ENST00000455855 | |  |
| Y | 6830000 | 7390000 | AMELY,ATP5PFP1,GOT2P5,GPR143P,PRKY | 560 | 0.76 | 1.52 |  | gain | 2.21E-153 | ENST00000651267, ENST00000215479, ENST00000383036 | |  |
| Y | 7390000 | 10200000 | AC064829.1,BPY2DP,CDC27P2,CDY3P,DUX4L31 | 2 810 | 0.8 | 1.6 | 0.884 | gain | 0 | ENST00000651511, ENST00000650816, ENST00000657784, ENST00000651700 | |  |
| Y | 11400000 | 12500000 | ARSDP1,ARSFP1,ARSLP1,ASS1P6,FAM8A4P | 1 100 | 0.771 | 1.54 | 0.89 | gain | 1.73E-205 | ENST00000443820 | |  |
| Y | 12500000 | 12600000 | SHROOM2P1,USP9Y | 100 | 0.96 | 1.92 |  | gain | 0.0000011 | ENST00000434773 | |  |
| Y | 12600000 | 13500000 | AC244213.1,CASKP1,CDY4P,DDX3Y,DPPA2P1 | 900 | 0.842 | 1.68 | 0.807 | gain | 9.77E-144 | ENST00000652216, ENST00000662422, ENST00000651143, ENST00000656670 | |  |
| Y | 13500000 | 15000000 | AC006989.1,AC010723.1,AGKP1,ANOS2P,CYCSP46 | 1 500 | 0.802 | 1.6 | 0.934 | gain | 9.29E-293 | ENST00000427212 | |  |
| Y | 15000000 | 15200000 |  | 200 | 0.827 | 1.65 |  | gain | 1.22E-48 |  | |  |
| Y | 15200000 | 15300000 |  | 100 | 0.833 | 1.67 |  | gain | 5.07E-21 |  | |  |
| Y | 15300000 | 17300000 | AC011749.1,AC011751.1,PUDPP1,RNU6,STSP1 | 2 000 | 0.793 | 1.59 | 0.872 | gain | 0 | ENST00000516880 | |  |
| Y | 17300000 | 18900000 | ACTG1P11,ACTG1P2,CDY2A,CDY2B,CDY5P | 1 600 | 0.798 | 1.6 |  | gain | 1.24E-45 | ENST00000425411 | |  |
| Y | 18900000 | 19900000 | AC010889.2,BCORP1,CD24P4,GAPDHP19,KDM5D | 1 000 | 0.807 | 1.61 | 0.851 | gain | 1.82E-215 | ENST00000653234, ENST00000652994 | |  |
| Y | 19900000 | 20400000 | RCC2P2,ZNF885P,ZNF886P | 500 | 0.88 | 1.76 | 0.881 | gain | 9.97E-10 | ENST00000422174 | |  |
| Y | 20400000 | 21600000 | AC010141.2,CDY10P,EIF1AY,ELOCP5,GAPDHP17 | 1 200 | 0.82 | 1.64 | 0.853 | gain | 5.16E-197 | ENST00000456659 | |  |
| Y | 21600000 | 26600000 | ANKRD36P1,BPY2,BPY2B,BPY2C,CCNQP2 | 5 000 | 0.784 | 1.57 | 1 | gain | 1.44E-59 | ENST00000344424 | |  |
| Y | 26600000 | 57200000 | CTBP2P1 | 30 600 |  | -2 | 0.813 | gain | 1.84E-16 | ENST00000431853 | |  |
