# Supplementary material for: Diagnostic challenges in complicated case of glioblastoma
Source: Pathol Oncol Res. 2024 Oct 29;30:1611875. doi: 10.3389/pore.2024.1611875 (PMC11554483; doi:10.3389/pore.2024.1611875)
Supplement: Supplementary file 4 [file Table2.docx]

**Table S2: Copy number alterations detected with MLPA and MS/MLPA.** Blue bold marked letters – gain/amplification of probe detected beyond MLPA threshold (≥1.3), light blue marked letters – suspected gain of probe on/or just below threshold (1.15-1.29). Red bold marked letters – loss/deletion of probe beneath MLPA threshold (≤0.7), light red marked letters – suspected loss of probe on/or just above threshold (0.85-0.71).

| P370-BRAF-IDH1-IDH2 (number of probes) | **Gene-exon** | **Chr. Band** | **Probe target info** | **Score** |
| --- | --- | --- | --- | --- |
| SRGAP3-RAF1 fusion, 3p25 (n=5) | CRBN-10 | 3p26.3 | 03-003,169203 | 1.14 |
|  | SRGAP3-20 | 3p25.3 | 03-009,011163 | 0.90 |
|  | SRGAP3-2 | 3p25.3 | 03-009,141517 | 0.96 |
|  | RAF1-17 | 3p25.1 | 03-012,600310 | 0.97 |
|  | RAF1-2 | 3p25.1 | 03-012,635037 | 1.04 |
| MYB aberrations, 6q23.3 (n=6) | LAMA2-11 | 6q22.33 | 06-129,553121 | 0.98 |
|  | MYB-2 | 6q23.3 | 06-135,548740 | 0.94 |
|  | MYB-10 | 6q23.3 | 06-135,559958 | 1.00 |
|  | MYB-15 | 6q23.3 | 06-135,566026 | 0.98 |
|  | MYB-16 | 6q23.3 | 06-135,581243 | 0.92 |
|  | PLAGL1-7b | 6q24.2 | 06-144,323322 | 0.97 |
| KIAA1549-BRAF fusion, 7q34 (n=14) | IKZF1-2 | 7p12.2 | 07-050,329209 | **1.60** |
|  | SLC26A3-20 | 7q22.3 | 07-107,195138 | **1.78** |
|  | KIAA1549-19 | 7q34 | 07-138,175482 | **1.53** |
|  | KIAA1549-17 | 7q34 | 07-138,187498 | **1.71** |
|  | KIAA1549-11 | 7q34 | 07-138,216768 | **1.57** |
|  | KIAA1549-4 | 7q34 | 07-138,246445 | **1.74** |
|  | HIPK2-8 | 7q34 | 07-138,949673 | **1.68** |
|  | MKRN1-6 | 7q34 | 07-139,805474 | **1.72** |
|  | BRAF-17 | 7q34 | 07-140,086030 | **1.55** |
|  | BRAF-14 | 7q34 | 07-140,100413 | **1.93** |
|  | BRAF-12 | 7q34 | 07-140,124283 | **1.79** |
|  | BRAF-8 | 7q34 | 07-140,140688 | **1.90** |
|  | BRAF-4 | 7q34 | 07-140,155193 | **1.71** |
|  | CNTNAP2-4 | 7q35 | 07-146,371995 | **1.76** |
| FGFR1 and FGFR1-TACC1, 8p11-p12 (n=8) | FGFR1-18 | 8p12 | 08-038,389951 | 0.76 |
|  | FGFR1-14 | 8p12 | 08-038,391534 | 0.76 |
|  | FGFR1-13 | 8p12 | 08-038,392597 | 0.74 |
|  | FGFR1-10 | 8p12 | 08-038,394985 | 0.74 |
|  | FGFR1-5 | 8p12 | 08-038,404637 | **0.69** |
|  | FGFR1-2 | 8p12 | 08-038,434075 | 0.77 |
|  | TACC1-1 | 8p11.23 | 08-038,705072 | 0.73 |
|  | TACC1-11 | 8p11.23 | 08-038,818995 | 0.83 |
| MYBL1, 8q13.1 (n=4) | MYBL1-14 | 8q13.1 | 08-067,641447 | 0.87 |
|  | MYBL1-8 | 8q13.1 | 08-067,667201 | 0.81 |
|  | MYBL1-5 | 8q13.1 | 08-067,672137 | 0.81 |
|  | MYBL1-2 | 8q13.1 | 08-067,677227 | 0.80 |
| CDKN2A, CDKN2B and MIR31 genes, 9p21.3 (n=4) | MIR31-1 | 9p21.3 | 09-021,502096 | 1.16 |
|  | CDKN2A-4 | 9p21.3 | 09-021,958235 | 1.16 |
|  | CDKN2A-2 | 9p21.3 | 09-021,964958 | 1.16 |
|  | CDKN2B-down | 9p21.3 | 09-021,990531 | **1.45** |
| IDH1 R132H and R132C point mutations (n=2) | IDH1-6 (R132H) | 2q34 | 02-208,821301 | 0 |
|  | IDH1-6 (R132C) | 2q34 | 02-208,821328 | 0 |
| IDH2 R172M and R172K point mutations (n=2) | IDH2-5 (R172K) | 15q26.1 | 15-088,432808 | 0 |
|  | IDH2-5 (R172M) | 15q26.1 | 15-088,432808 | 0 |
| BRAF V600E point mutation (n=1) | BRAF-15 (V600E) | 7q34 | 07-140,099560 | 0 |
| P088-Oligodendroglioma 1p |  |  |  |  |
| 1p chromosome arm (n=19) | GNB1-3 | 1p36.33 | 01-001,746713 | 0.99 |
|  | TNFRSF14-8 | 1p36.32 | 01-002,479695 | 0.84 |
|  | TP73-1 | 1p36.32 | 01-003,558493 | **0.68** |
|  | TNFRSF9-2 | 1p36.23 | 01-007,923428 | 0.77 |
|  | MFN2-10 | 1p36.22 | 01-011,984395 | 0.93 |
|  | WNT4-2 | 1p36.12 | 01-022,328835 | 0.77 |
|  | PTAFR-3 | 1p35.3 | 01-028,349939 | 0.94 |
|  | MUTYH-9 | 1p34.1 | 01-045,570643 | 0.84 |
|  | PRDX1-2 | 1p34.1 | 01-045,760287 | 1.09 |
|  | FAF1-4 | 1p33 | 01-051,026388 | 0.71 |
|  | CDKN2C-1 | 1p33 | 01-051,207960 | 1.01 |
|  | CDKN2C-3 | 1p33 | 01-051,212265 | 1.10 |
|  | PLPP3-2 | 1p32.2 | 01-056,775291 | 0.99 |
|  | MIR101-1-1 | 1p31.3 | 01-065,296660 | 1.10 |
|  | FUBP1-8 | 1p31.1 | 01-078,203391 | 1.15 |
|  | GTF2B-2 | 1p22.2 | 01-089,125546 | 1.15 |
|  | DPYD-1 | 1p21.3 | 01-098,159098 | **1.31** |
|  | NRAS-5 | 1p13.2 | 01-115,052670 | 1.10 |
|  | NOTCH2-5 | 1p12 | 01-120,331104 | 1.21 |
| 1q chromosome arm (n=3) | LMNA-5 | 1q22 | 01-154,371571 | 0.90 |
|  | CRB1-5 | 1q31.3 | 01-195,592638 | 1.10 |
|  | TNNT2-7 | 1q32.1 | 01-199,603509 | 0.73 |
| CDKN2A, CDKN2B genes, 9p21.3 (n=5) | CDKN2A-3 | 9p21.3 | 09-021,961213 | 1.13 |
|  | CDKN2A-2 | 9p21.3 | 09-021,964957 | 1.22 |
|  | CDKN2A-1 | 9p21.3 | 09-021,984375 | **1.37** |
|  | CDKN2B-2 | 9p21.3 | 09-021,995813 | **1.36** |
|  | CDKN2B-1 | 9p21.3 | 09-021,998949 | **1.44** |
| 19p chromosome arm (n=2) | SMARCA4-35 | 19p13.2 | 19-011,031428 | 0.82 |
|  | LDLR-4 | 19p13.2 | 19-011,076948 | 0.89 |
| 19q chromosome arm (n=12) | CCNE1-11 | 19q12 | 19-035,005212 | 0.81 |
|  | PDCD5-1 | 19q13.11 | 19-037,764006 | 1.14 |
|  | SLC7A9-13 | 19q13.11 | 19-038,013357 | 0.83 |
|  | UPK1A-4 | 19q13.12 | 19-040,856158 | 0.80 |
|  | WDR62-31 | 19q13.12 | 19-041,287360 | 0.73 |
|  | TGFB1-3 | 19q13.2 | 19-046,542497 | 0.85 |
|  | CIC-10 | 19q13.2 | 19-047,486622 | 0.83 |
|  | ZNF296-2 | 19q13.32 | 19-050,270824 | 0.89 |
|  | PPP1R15A-2 | 19q13.33 | 19-054,069622 | **1.35** |
|  | BAX-4 | 19q13.33 | 19-054,151278 | 1.07 |
|  | CHMP2A-4 | 19q13.43 | 19-063,755278 | 1.06 |
|  | CHMP2A-2 | 19q13.43 | 19-063,757281 | 1.26 |
| IDH1 R132H and R132C point mutations (n=2) | IDH1-6 ( R132H) | 2q34 | 02-208,821301 | 0 |
|  | IDH1-6 ( R132C) | 2q34 | 02-208,821328 | 0 |
| IDH2 R172M and R172K point mutations (n=2) | IDH2-5 ( R172K) | 15q26.1 | 15-088,432808 | 0 |
|  | IDH2-5 ( R172M) | 15q26.1 | 15-088,432808 | 0 |
| P105-Glioma2 |  |  |  |  |
| PDGFRA gene, 4q12 (n=3) | PDGFRA-3 | 4q12 | 04-054,822105 | 1.07 |
|  | PDGFRA-5 | 4q12 | 04-054,825892 | 1.04 |
|  | PDGFRA-22 | 4q12 | 04-054,851258 | 1.02 |
| EGFR gene, 7p11.2 (n=11) | EGFR-1 | 7p11.2 | 07-055,054393 | **1.73** |
|  | EGFR-2 | 7p11.2 | 07-055,177534 | **1.79** |
|  | EGFR-3 | 7p11.2 | 07-055,178494 | **1.75** |
|  | EGFR-4 | 7p11.2 | 07-055,181895 | **1.63** |
|  | EGFR-5 | 7p11.2 | 07-055,186485 | **1.68** |
|  | EGFR-6 | 7p11.2 | 07-055,187732 | **1.62** |
|  | EGFR-7 | 7p11.2 | 07-055,189281 | **1.60** |
|  | EGFR-8 | 7p11.2 | 07-055,191052 | **1.68** |
|  | EGFR-13 | 7p11.2 | 07-055,196768 | **1.63** |
|  | EGFR-16 | 7p11.2 | 07-055,206352 | **1.66** |
|  | EGFR-23 | 7p11.2 | 07-055,233952 | **1.70** |
| CDKN2A gene, 9p21.3 (n=4) | CDKN2A-4 | 9p21.3 | 09-021,957858 | 1.17 |
|  | CDKN2A-3 | 9p21.3 | 09-021,960899 | 1.20 |
|  | CDKN2A-2 | 9p21.3 | 09-021,964957 | 1.16 |
|  | CDKN2A-1 | 9p21.3 | 09-021,984532 | **1.30** |
| PTEN gene, 10q23.31 (n=9) | PTEN-1 | 10q23.31 | 10-089,614104 | 0.94 |
|  | PTEN-2 | 10q23.31 | 10-089,644014 | 0.92 |
|  | PTEN-3 | 10q23.31 | 10-089,675492 | 0.88 |
|  | PTEN-4 | 10q23.31 | 10-089,680713 | 0.92 |
|  | PTEN-5 | 10q23.31 | 10-089,682858 | 0.97 |
|  | PTEN-6 | 10q23.31 | 10-089,701944 | 0.90 |
|  | PTEN-7 | 10q23.31 | 10-089,707626 | 0.96 |
|  | PTEN-8 | 10q23.31 | 10-089,710740 | 1.01 |
|  | PTEN-9 | 10q23.31 | 10-089,716114 | 0.91 |
| CDK4, MIR26A2 and MDM2 genes, 12q14.1-q15 (n=5) | CDK4-8 | 12q14.1 | 12-056,428450 | 1.07 |
|  | CDK4-4 | 12q14.1 | 12-056,430960 | 1.04 |
|  | MIR26A2-1 | 12q14.1 | 12-056,504665 | 0.94 |
|  | MDM2-2 | 12q15 | 12-067,489255 | 1.23 |
|  | MDM2-10 | 12q15 | 12-067,516726 | 1.22 |
| NFKBIA gene, 14q13.2 (n=3) | NFKBIA-5 | 14q13.2 | 14-034,941523 | 0.87 |
|  | NFKBIA-4 | 14q13.2 | 14-034,941720 | 0.94 |
|  | NFKBIA-3 | 14q13.2 | 14-034,942273 | 0.97 |
| TP53 gene, 17p13.1 (n=8) | TP53-11 | 17p13.1 | 17-007,513641 | 0.73 |
|  | TP53-10 | 17p13.1 | 17-007,514674 | 0.76 |
|  | TP53-7 | 17p13.1 | 17-007,517784 | 0.75 |
|  | TP53-5 | 17p13.1 | 17-007,518934 | 0.71 |
|  | TP53-4b | 17p13.1 | 17-007,519217 | 0.74 |
|  | TP53-3 | 17p13.1 | 17-007,520063 | 0.77 |
|  | TP53-2a | 17p13.1 | 17-007,520626 | 0.74 |
|  | TP53-1 | 17p13.1 | 17-007,531444 | 0.94 |
| ME012-MGMT-IDH1-IDH2 |  |  |  |  |
| MGMT gene, 10q26.3 (n=6) | MGMT-1 HhaI | 10q26.3 | 10-131,155090 | **0.69** |
|  | MGMT-1 HhaI | 10q26.3 | 10-131,155200 | **0.68** |
|  | MGMT-1 HhaI | 10q26.3 | 10-131,155610 | **0.66** |
|  | MGMT-1 HhaI | 10q26.3 | 10-131,155690 | **0.70** |
|  | MGMT-1 HhaI | 10q26.3 | 10-131,155020 | **0.69** |
|  | MGMT-1 HhaI | 10q26.3 | 10-131,155480 | **0.66** |
| IDH1 R132H and R132C point mutations (n=2) | IDH1-6 (R132H) | 2q34 | 02-208,821320 | 0 |
|  | IDH1-6 (R132C) | 2q34 | 02-208,821340 | 0 |
| IDH2 R172M and R172K point mutations (n=2) | IDH2-5 (R172K) | 15q26.1 | 15-088,432820 | 0 |
|  | IDH2-5 (R172M) | 15q26.1 | 15-088,432820 | 0 |
