# Supplementary material for: Diagnostic challenges in complicated case of glioblastoma
Source: Pathol Oncol Res. 2024 Oct 29;30:1611875. doi: 10.3389/pore.2024.1611875 (PMC11554483; doi:10.3389/pore.2024.1611875)
Supplement: Supplementary file 5 [file Table5.docx]

**Table S5: List of CNAs detected with gene panel.** Chr – chromosome, CN - Relative copy number (NOT log-2). A copy number of 1 means normal diploid , P Val –The result of a two-tailed Wilcoxon rank sum test; null hypothesis is that the median value of the copy number called for each probe of the given group is equal to the median value of the copy number called for all primers identified as being in the baseline., GSP - number of GSPs supporting the CNA event; Rel - Relevance classification of the CNA: Gain or Loss for full group event, and Partial Gain or Partial Loss for sub-group events.

| **Gene** | **Chr** | **CN** | **P Val** | **Transcript** | **GSPs** | **Rel** |
| --- | --- | --- | --- | --- | --- | --- |
| *AURKA* | 20 | 1.393 | 0.00111 | NM_003600.4 | 6 | Gain |
| *EGFR* | 7 | 1.579 | 0.00058 | NM_005228.5 | 6 | Gain |
| *FGFR1* | 8 | 0.606 | 0.00111 | NM_015850.4 | 6 | Loss |
| *FGFR2* | 10 | 0.683 | 0.00339 | NM_000141.4 | 7 | Loss |
| *FGFR3* | 4 | 0.696 | 0.00505 | NM_000142.4 | 7 | Loss |
| *FLT3* | 13 | 0.674 | 0.00411 | NM_004119.3 | 6 | Loss |
| *GNAS* | 20 | 1.548 | 0.00058 | NM_000516.6 | 6 | Gain |
| *JAK2* | 9 | 1.269 | 0.00646 | NM_004972.3 | 6 | Gain |
| *MET* | 7 | 2.036 | 0.00040 | NM_000245.4 | 6 | Gain |
| *MYC* | 8 | 0.728 | 0.01475 | NM_002467.6 | 6 | Loss |
| *RET* | 10 | 0.575 | 0.00014 | NM_020630.5 | 8 | Loss |
| *SMAD4* | 18 | 1.311 | 0.00275 | NM_005359 | 4 | Partial Gain |
| *SMARCB1* | 22 | 0.632 | 0.00131 | NM_003073.5 | 6 | Loss |
| *SMO* | 7 | 1.432 | 0.00111 | NM_005631.5 | 6 | Gain |
| *TP53* | 17 | 0.632 | 0.00011 | NM_000546.5 | 11 | Loss |
