# Supplementary material for: Diagnostic challenges in complicated case of glioblastoma
Source: Pathol Oncol Res. 2024 Oct 29;30:1611875. doi: 10.3389/pore.2024.1611875 (PMC11554483; doi:10.3389/pore.2024.1611875)
Supplement: Supplementary file 6 [file Table3.docx]

**Table S3: Copy number alterations detected with aCGH/SNP.** Chr – chromosome, Mean Log ration - Mean Logaritmic Ratio , ISCN - The International System for Human Cytogenomic Nomenclature, kb – kilo base pairs.

| Chr | Start | Stop | Cytoband | Size (kb) | Type | #Probes | Mean log ratio | ISCN | |
| --- | --- | --- | --- | --- | --- | --- | --- | --- | --- |
| 1 | 3386390 | 8495600 | p36.32 - p36.23 | 5109.2 | Loss | 227 | -0.41 | arr[GRCh37] 1p36.32p36.23(3386390_8495600)x1 |  |
| 1 | 8512670 | 8747793 | p36.23 | 235.1 | Loss | 10 | -1.40 | arr[GRCh37] 1p36.23(8512670_8747793)x1 |  |
| 1 | 8803013 | 11052701 | p36.23 - p36.22 | 2249.7 | Loss | 91 | -0.45 | arr[GRCh37] 1p36.23p36.22(8803013_11052701)x1 |  |
| 1 | 104480522 | 108146890 | p21.1 - p13.3 | 3666.4 | Gain | 130 | 0.30 | arr[GRCh37] 1p21.1p13.3(104480522_108146890)x2~3 |  |
| 2 | 145215331 | 149246221 | q22.3 - q23.1 | 4030.9 | Gain | 153 | 0.41 | arr[GRCh37] 2q22.3q23.1(145215331_149246221)x2~3 |  |
| 2 | 154044207 | 163823050 | q23.3 - q24.3 | 9778.8 | Gain | 345 | 0.42 | arr[GRCh37] 2q23.3q24.3(154044207_163823050)x3 |  |
| 6 | 104521599 | 170921089 | q16.3 - q27 | 66399.5 | Loss | 2562 | -0.15 | arr[GRCh37] 6q16.3q27(104521599_170921089)x1 |  |
| 7 | 58764 | 57642437 | p22.3 - p11.2 | 57583.7 | Gain | 2208 | 0.78 | arr[GRCh37] 7p22.3p11.2(58764_57642437)x4~5 |  |
| 7 | 61831840 | 159118566 | q11.21 - q36.3 | 97286.7 | Gain | 3749 | 0.76 | arr[GRCh37] 7q11.21q36.3(61831840_159118566)x4~5 |  |
| 8 | 47843 | 43819937 | p23.3 - p11.1 | 43772.1 | Loss | 1740 | -0.41 | arr[GRCh37] 8p23.3p11.1(47843_43819937)x1 |  |
| 8 | 46943457 | 146293435 | q11.1 - q24.3 | 99350.0 | Loss | 3797 | -0.40 | arr[GRCh37] 8q11.1q24.3(46943457_146293435)x1 |  |
| 9 | 8313246 | 14542140 | p24.1 - p22.3 | 6228.9 | Gain | 209 | 0.26 | arr[GRCh37] 9p24.1p22.3(8313246_14542140)x2~3 |  |
| 9 | 23933298 | 31607144 | p21.3p21.1 | 7673.8 | Gain | 275 | 0.23 | arr[GRCh37] 9p21.3p21.1(23933298_31607144)x3 |  |
| 10 | 102539 | 39047586 | p15.3 - p11.1 | 38945.0 | Loss | 1483 | -0.44 | arr[GRCh37] 10p15.3p11.1(102539_39047586)x1 |  |
| 10 | 42720738 | 135434178 | q11.21 - q26.3 | 92713.4 | Loss | 3470 | -0.43 | arr[GRCh37] 10q11.21q26.3(42720738_135434178)x1 |  |
| 13 | 19024748 | 115107245 | q11 - q34 | 96082.5 | Loss | 3601 | -0.37 | arr[GRCh37] 13q11q34(19024748_115107245)x1 |  |
| 14 | 19100682 | 106327993 | q11.2 - q32.33 | 87227.3 | Loss | 3490 | -0.43 | arr[GRCh37] 14q11.2q32.33(19100682_106327993)x1 |  |
| 17 | 5390784 | 7720460 | p13.2 - p13.1 | 2329.7 | Loss | 95 | -0.50 | arr[GRCh37] 17p13.2p13.1(5390784_7720460)x1 |  |
| 17 | 25343161 | 29497017 | q11.1 - q12 | 4153.9 | Loss | 157 | -0.49 | arr[GRCh37] 17q11.1q11.2(25343161_29497017)x1 |  |
| 17 | 29739462 | 32525914 | q11.2 - q12 | 2786.5 | Loss | 110 | -0.50 | arr[GRCh37] 17q11.2q12(29739462_32525914)x1 |  |
| 18 | 26228320 | 28170500 | q12.1 | 1942.2 | Gain | 70 | 0.33 | arr[GRCh37] 18q12.1(26228320_28170500)x2~3 |  |
| 18 | 62137385 | 72481634 | q22.1 - q22.3 | 10344.3 | Gain | 358 | 0.27 | arr[GRCh37] 18q22.1q22.3(62137385_72481634)x2~3 |  |
| 20 | 61012 | 25519079 | p13 - p11.21 | 25458.1 | Gain | 920 | 0.44 | arr[GRCh37] 20p13p11.21(61012_25519079)x3 |  |
| 20 | 29423251 | 32837328 | q11.21 - q11.22 | 3414.1 | Gain | 136 | 0.30 | arr[GRCh37] 20q11.21q11.22(29423251_32837328)x2~3 |  |
| 20 | 49378646 | 50686696 | q13.13 - q13.2 | 1308.1 | Gain | 52 | 0.34 | arr[GRCh37] 20q13.13q13.2(49378646_50686696)x2~3 |  |
| 20 | 54568755 | 61723009 | q13.2 - q13.33 | 7154.3 | Gain | 326 | 0.40 | arr[GRCh37] 20q13.2q13.33(54568755_61723009)x3 |  |
| 22 | 16133474 | 51224252 | q11.1 - q13.33 | 35090.8 | Loss | 1702 | -0.46 | arr[GRCh37] 22q11.1q13.33(16133474_51224252)x1 |  |
| Y | 95768 | 9901780 | p11.32 - p11.2 | 9806.0 | Gain | 311 | 0.54 | arr[GRCh37] Yp11.32p11.2(95768_2699520x3.2699521_9901780x2) |  |
| Y | 13872502 | 59214589 | q11.21 - q12 | 45342.1 | Gain | 340 | 0.59 | arr[GRCh37] Yq11.21q12(13872502_59214589)x2 |  |
|  |  |  |  |  |  |  |  |  |  |
