# Supplementary material for: Diagnostic challenges in complicated case of glioblastoma
Source: Pathol Oncol Res. 2024 Oct 29;30:1611875. doi: 10.3389/pore.2024.1611875 (PMC11554483; doi:10.3389/pore.2024.1611875)

**Figure S2: Genomic landscape of SVs and CNAs in a human glioblastoma sample.** The plot consists of five layers of data. Starting outward, the ideogram portrays the human genome (GRCh38). Moving inward, two layers showcase copy number (CN) and B-allele frequency (BAF) values from ASCAT, providing essential insights for understanding CNVs. The first layer shows copy numbers on a normalized logarithmic scale, where gains are red (CN > 2.55), losses are blue (CN < 1.41) and the normal state (CNA = [1.41, 2.55]) is grey. The second layer shows B-allele frequency (BAF). In diploid samples, there are three possibilities: reference homozygous (BAF = 0), heterozygous (BAF = 0.5), or variant homozygous (BAF = 1 Notably, chromosomes 8, 10, 13, 14, and 22 display loss of heterozygosity (LOH), indicated by a distinct pattern. Additionally, the LOH events are also observable in the ASCAT plot (Figure S1), where one allele is entirely lost. The penultimate inner layer depicts intrachromosomal SVs, marking deletions (blue), duplications (red), insertions (green), and translocations (grey). The innermost layer highlights interchromosomal SVs (black).


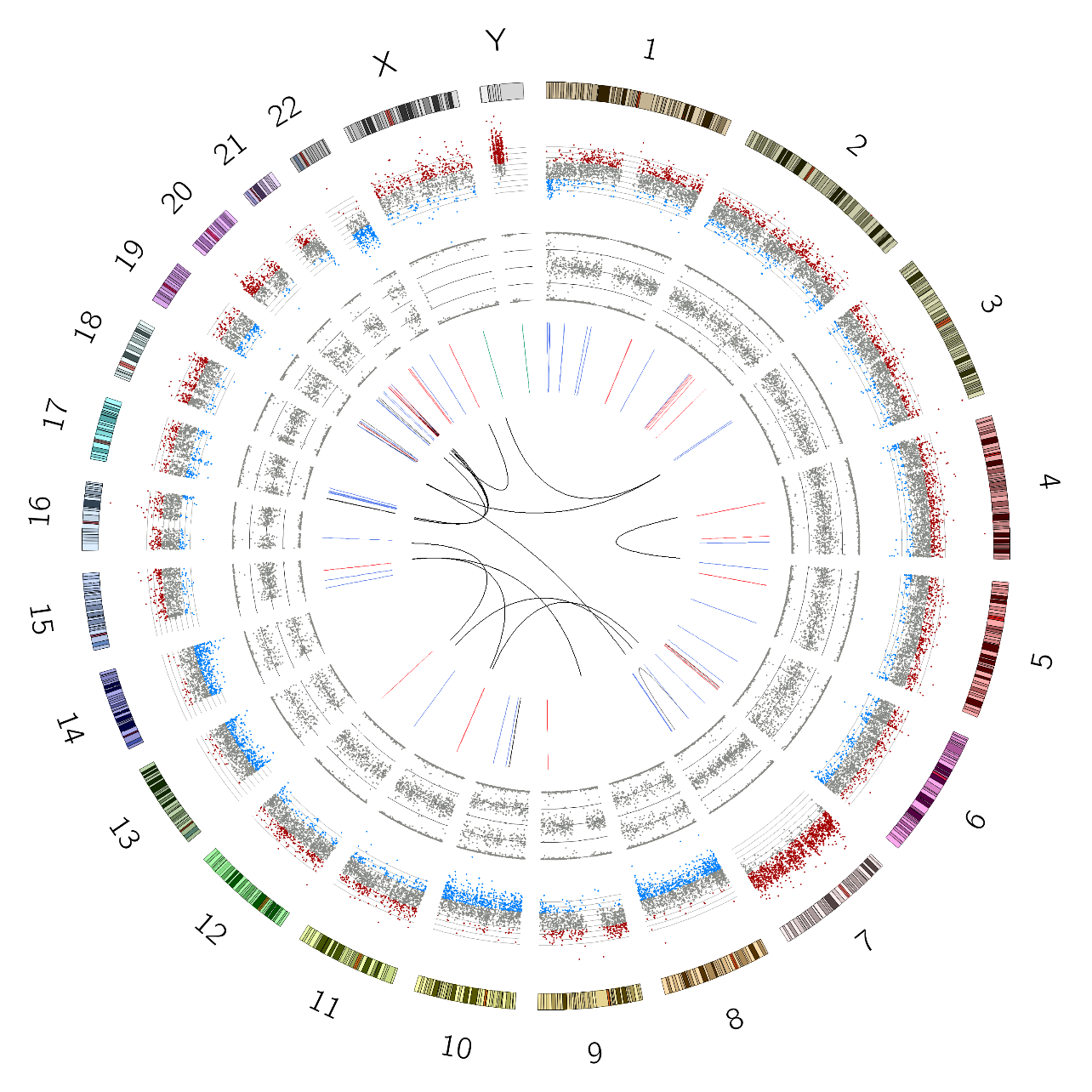

Supplement: Supplementary file 7 [file DataSheet2.docx]
