# Supplementary material for: Diagnostic challenges in complicated case of glioblastoma
Source: Pathol Oncol Res. 2024 Oct 29;30:1611875. doi: 10.3389/pore.2024.1611875 (PMC11554483; doi:10.3389/pore.2024.1611875)
Supplement: Supplementary file 8 [file Table4.docx]

**Table S4: List of mutations detected with gene panels in samples of glioblastoma and peripheral blood.** HGVSp – Human Genome Variant society protein description, HGVSc = Human Variant society cDNA description, Tier – interpretation and scoring system of somatic variant (Li et al., 2017), Tier I - variants with strong clinical significance, Tier IV - variants benign or likely benign; VAF – variant allele frequency, gnomAD AF – allele frequency in European (non-Finnish) population, rs - reference identifier.

| Gene | HGVSp | HGVSc | Tier | Glioblastoma | | Peripheral blood | | | gnomAD AF | COSMICID | rs | Consequence | Transcript | Genomic Location |
| --- | --- | --- | --- | --- | --- | --- | --- | --- | --- | --- | --- | --- | --- | --- |
|  |  |  |  | **VAF** |  | **VAF** | |  |  |  |  |  |  |  |
| *ATM* | F858L | c.2572T>C | IV | 0.4 |  | 0.5 | |  | 0.008650 | COSM21826 | rs1800056 | missense variant | NM_000051.4 | chr11:108138003 |
| *CDH1* | A102S | c.304G>T | IV | 0.5 | | 0.5 | |  | 0,000008 |  | rs368492235 | missense variant | NM_004360.5 | chr16:68835713 |
| *ERBB4* |  | c.884-8_884-7del | IV | 0.2 |  | 0.2 | |  | 0.027990 |  | rs67894136 | splice region variant & splice polypyrimidine tract variant & intron variant | NM_005235.3 | chr2:212578379 |
| *ERBB4* |  | c.884-9_884-7del | IV | 0.1 | | 0.1 | |  | 0.000335 |  | rs67894136 | splice region variant & splice polypyrimidine tract variant & intron variant | NM_005235.3 | chr2:212578379 |
| ***TP53*** | **R196*** | **c.586C>T** | **I** | **0.5** |  |  |  |  | **0.000004** | **COSM99666** | **rs397516435** | **stop gained** | **NM_000546.6** | **chr17:7578263** |
