# Supplementary material for: Diagnostic challenges in complicated case of glioblastoma
Source: Pathol Oncol Res. 2024 Oct 29;30:1611875. doi: 10.3389/pore.2024.1611875 (PMC11554483; doi:10.3389/pore.2024.1611875)
Supplement: Supplementary file 9 [file Table6.docx]

**Table S6: List of mutations detected with WGS.** Chr – chromosome, HGVSp – Human Genome Variant society protein description, HGVSc = Human Variant society cDNA description, Tier – interpretation and scoring system of somatic variant (Li et al., 2017), Tier I - variants with strong clinical significance, Tier II - variants with potential clinical significance, Tier III - variants of unknown clinical significance, Tier IV - variants benign or likely benign; VAF – variant allele frequency, Effect - Ensembl variant effect predictor (VEP) the effects of genomic variation on genome function and how these effects shape phenotypes, Featureid – Ensemble gene annotation including any spliced transcripts, Impact - predicted impact of the variants on genome.

| Chr | Gene | HGVSp | HGVSc | VAF (%) | Tier | Effect | Genomic location | Transcript | Featureid | Impact |
| --- | --- | --- | --- | --- | --- | --- | --- | --- | --- | --- |
| 20 | *TOP1* | p.Lys194Glu | c.580A>G | 12,04 | III | missense variant | chr20:39713174 | NM_003286 | ENST00000361337.2 | MODERATE |
| 19 | ***SYDE1*** | **p.Trp83*** | **c.248G>A** | **13,71** | **II** | **stop gained** | **chr19:15220026** | **NM_033025** | **ENST00000342784.6** | **HIGH** |
| 19 | ***ZNF567*** | **p.His253fs** | **c.757delC** | **16,73** | **II** | **frameshift variant** | **chr19:37210382** | **NM_001387766, NM_001387769, NM_001387767** | **ENST00000536254.6** | **HIGH** |
| 16 | *XPO6* | p.Thr1098Ala | c.3292A>G | 30,13 | III | missense variant | chr16:28109945 | NM_015171 | ENST00000304658.9 | MODERATE |
| 17 | *TTC25* | p.Glu19Lys | c.55G>A | 30,16 | III | missense variant | chr17:40087031 | NM_031421, NM_001350319 | ENST00000377540.5 | MODERATE |
| 17 | ***TP53*** | **p.Arg157*** | **c.469C>T** | **30,35** | **I** | **stop gained** | **chr17:7578263** |  | **ENST00000635293.1** | **HIGH** |
| 19 | *ZNF98* | p.Pro321His | c.962C>A | 30,51 | III | missense variant | chr19:22575075 | NM_001098626 | ENST00000357774.9 | MODERATE |
| 12 | *DUSP6* |  | c.871G>C | 30,7 | III | structural interaction variant | chr12:89743306 | NM_001946 | ENST00000279488 | HIGH |
| 12 | *DUSP6* | p.Val291Leu | c.871G>C | 30,7 | III | missense variant | chr12:89743306 | NM_001946 | ENST00000279488.7 | MODERATE |
| 3 | *SLC12A8* | p.Met652Leu | c.1954A>T | 30,93 | III | missense variant | chr3:124807182 | NM_001195483 | ENST00000393469.8 | MODERATE |
| 4 | ***COQ2*** | **p.Lys190fs** | **c.569delA** | **31,33** | **II** | **frameshift variant & splice region variant** | **chr4:84200101** | **NM_015697** | **ENST00000311469.8** | **HIGH** |
| 9 | *GRHPR* | p.Gly148Ser | c.442G>A | 33,32 | III | missense variant | chr9:37428518 |  | ENST00000607784.1 | MODERATE |
| 5 | *GABRA1* | p.Val89Ile | c.265G>A | 35,26 | III | missense variant | chr5:161292759 |  | ENST00000638159.1 | MODERATE |
| 21 | *CHODL* | p.Tyr148Cys | c.443A>G | 40,11 | III | missense variant | chr21:19629340 | NM_024944 | ENST00000299295.6 | MODERATE |
| 1 | ***ACTRT2*** | **p.Trp96*** | **c.288G>A** | **41,01** | **II** | **stop gained** | **chr1:2938538** | **NM_080431** | **ENST00000378404.3** | **HIGH** |
| 16 | *DNAJA2* | p.Val312Leu | c.934G>C | 41,37 | III | missense variant | chr16:46993028 | NM_005880 | ENST00000317089.9 | MODERATE |
| 5 | *SV2C* | p.Thr696Pro | c.2086A>C | 41,45 | III | missense variant | chr5:75621274 | NM_014979 | ENST00000502798.6 | MODERATE |
| 6 | *DNAH8* | p.Met2735Ile | c.8205G>A | 42,81 | III | missense variant | chr6:38851720 | NM_001206927 | ENST00000327475.10 | MODERATE |
| 13 | ***RB1*** | **p.Ser215*** | **c.644C>A** | **47,85** | **II** | **stop gained** | **chr13:48934189** | **NM_000321** | **ENST00000267163.4** | **HIGH** |
| X | ***MSL3*** | **p.Arg228fs** | **c.684delG** | **48,04** | **II** | **frameshift variant** | **chrX:11781049** | **NM_078629** | **ENST00000312196.8** | **HIGH** |
