# Supplementary material for: Diagnostic challenges in complicated case of glioblastoma
Source: Pathol Oncol Res. 2024 Oct 29;30:1611875. doi: 10.3389/pore.2024.1611875 (PMC11554483; doi:10.3389/pore.2024.1611875)

**Figure S1: ASCAT profile.** The plot depicts a genome-wide allele-specific copy number for all heterozygous assayed loci in a high-grade human glioblastoma sample. It visually represents gains/amplifications, loss/deletions, loss of heterozygosity (LOH), and copy-neutral events. The red line corresponds to the major allele, and the green line corresponds to the minor allele. The lines are shifted to avoid overlapping. The sample is a near-tetraploid tumor sample (ploidy = 3.83) with an aberrant cell fraction of 46%. The goodness of fit measures the degree to which the chosen values for tumor ploidy and non-aberrant cell admixture align with the dataset.


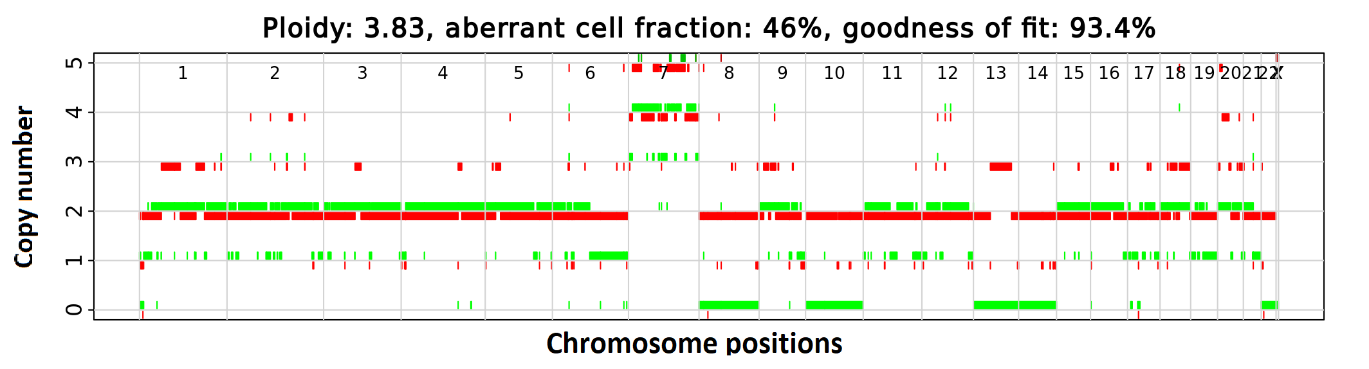

Supplement: Supplementary file 10 [file DataSheet1.docx]
